# Developing an ontology of non-pharmacological treatment for emotional and mood disturbances in dementia

**DOI:** 10.1038/s41598-023-46226-5

**Published:** 2024-01-22

**Authors:** Zhenyu Zhang, Ping Yu, Mengyang Yin, Hui Chen Chang, Susan J. Thomas, Wenxi Wei, Ting Song, Chao Deng

**Affiliations:** 1 Centre for Digital Transformation, School of Computing and Information Technology, University of Wollongong, Northfield Ave, Wollongong, NSW 2522 Australia; 2 Illawarra Health and Medical Research Institute, University of Wollongong, Wollongong, Australia; 3 Systems and Reporting Residential Care, Catholic Healthcare Ltd, Wollongong, Australia; 4 School of Nursing, University of Wollongong, Wollongong, Australia; 5 Graduate School of Medicine, University of Wollongong, Wollongong, Australia; 6 School of Medical, Indigenous and Health Sciences, University of Wollongong, Wollongong, Australia

**Keywords:** Computational biology and bioinformatics, Health care

## Abstract

Emotional and mood disturbances are common in people with dementia. Non-pharmacological interventions are beneficial for managing these disturbances. However, effectively applying these interventions, particularly in the person-centred approach, is a complex and knowledge-intensive task. Healthcare professionals need the assistance of tools to obtain all relevant information that is often buried in a vast amount of clinical data to form a holistic understanding of the person for successfully applying non-pharmacological interventions. A machine-readable knowledge model, e.g., ontology, can codify the research evidence to underpin these tools. For the first time, this study aims to develop an ontology entitled Dementia-Related Emotional And Mood Disturbance Non-Pharmacological Treatment Ontology (DREAMDNPTO). DREAMDNPTO consists of 1258 unique classes (concepts) and 70 object properties that represent relationships between these classes. It meets the requirements and quality standards for biomedical ontology. As DREAMDNPTO provides a computerisable semantic representation of knowledge specific to non-pharmacological treatment for emotional and mood disturbances in dementia, it will facilitate the application of machine learning to this particular and important health domain of emotional and mood disturbance management for people with dementia.

## Introduction

Dementia is a common illness among older people. In 2020, over 55 million people worldwide lived with dementia ^1^. This figure is projected to rise to 139 million by 2050 due to the ageing population and increasing life expectancy ^1^. People with dementia live with the condition for an average of 5–10 years, depending on the type of dementia ^2^. People’s memory, cognition, and ability to perform the activities of daily living are significantly affected by the disease, resulting in a reduced quality of life and high dependency on others for care ^1^. The annual cost of dementia care exceeds 1.3 trillion U.S. dollars, representing 1.53% of global GDP in 2021 ^1^. As a result, dementia care has emerged as a priority for the health and aged care sectors; this has posed an enormous social and economic burden on the person, family, health care system and society ^3,4^.

Due to the lack of clear pathophysiology or cure, supporting people to live well with dementia remains the focus of dementia care ^5,6^. One of the main challenges is the management of behavioural and psychological symptoms of dementia (BPSD) ^5,6^. These include agitated behaviours, psychotic symptoms (delusions and hallucinations), and emotional and mood disturbances (e.g., apathy, anxiety, and depression) ^7^. Emotional and mood disturbances prevail in about 90% of people with dementia ^8,9^. These disturbances can cause distress and harm for people with dementia, their families and caregivers, increased hospitalisation, increased residential care placement, and use of healthcare resources ^8,10^; therefore, managing these disturbances is a key dementia care task.

The emotional and mood disturbances can be treated with both pharmacological interventions (e.g., antipsychotics and antidepressants) and non-pharmacological interventions (e.g., music and massage) ^11,12^. Given the serious side effects of pharmacological interventions (e.g., metabolic disorders, extrapyramidal symptoms and dizziness that may lead to falls in people with dementia) ^11,13–15^, many authoritative healthcare organisations, such as the American Geriatrics Society and the U.K. National Institute for Health and Care Excellence recommend using non-pharmacological interventions as the first-line treatment approach ^16–19^, given the successful evidence of this approach delivered in person-centred care ^5,8,20^.

Managing emotional and mood disturbances in dementia with a person-centred non-pharmacological approach is a complex and knowledge-intensive task ^6^. It requires healthcare professionals to have an in-depth knowledge of various non-pharmacological interventions and a thorough understanding of the life history, health conditions, and preferences of the person with the symptom ^21,22^. To date, the relevant information is often buried in an obscuring mass of heterogeneous and unstructured clinical data ^23^, which is time-consuming to search and process manually. This limits the ability of healthcare professionals to effectively access and integrate the relevant data to fully understand the person for applying non-pharmacological interventions successfully. This limitation results in the ineffective implementation, or “trial-and-error” approaches to identifying effective interventions ^24^, and increased dependence on medications to manage the emotional and mood disturbances in people with dementia ^25^.

Knowledge graph is a type of artificial intelligence (AI) technology that captures and represents knowledge as a graph of entities and their relationships ^26^. Google first coined the term. Many companies, e.g., Google, AirBnB, eBay, Elsevier, Facebook, Microsoft and Springer Nature, have announced using a knowledge graph to manage information assets. A knowledge graph is promising in helping healthcare professionals to access and integrate relevant data for person-entered dementia care. First, it can integrate structured and unstructured data from electronic health records (EHR), medication histories, laboratory tests, and social care assessments ^27^. Second, it can facilitate automatic extraction and linkage of relevant information from multiple sources ^28^. These will enable healthcare professionals to access comprehensive information about a person with dementia, help them to identify critical data points and acquire a comprehensive view of the needs of a person with dementia. This broad view will enable healthcare professionals to tailor their care plans to the needs of the person with dementia. Knowledge graph can also facilitate integrating data from heterogeneous data sources ^29^ to enable multiple healthcare professionals to access the same client information and work together towards a common goal.

A knowledge graph is a type of database that uses ontologies to structure and organize its knowledge ^30^. Ontology is “a formal explicit specification of shared conceptualization” ^31^. It represents the structure of the knowledge graph, its entities and their relationships. For example, an ontology for nursing care can include concepts such as nursing assessment, care plan, care action, and care evaluation.

Ontology applies many semantic technologies, e.g., the Resource Description Framework (RDF) ^32^ and descriptive logics ^33^, to represent knowledge in a formal semantic web language called Web Ontology Language (OWL) ^32^. These technologies enable ontology to give meaning to data that machines can understand. Thus, instead of processing the data by just matching words/characters, machines can process data based on their meaning and logical relationships. Ontology can solve the problem of semantic heterogeneity (i.e., differences in the meaning of words), improve information retrieval and manipulation, and facilitate knowledge sharing and reuse ^34^. It plays a crucial role in semantic data management, semantic data storage, semantic query, and semantic extension ^34^.

Since the early 1990s, ontology has been a popular topic in many research communities, e.g., knowledge engineering, machine learning, and natural language processing ^35^. Moreover, ontology has been widely applied to many domains, such as health, finance, and geology ^35^. In the past 40 years, there has been increasing use of ontologies to represent knowledge in the health domain, e.g., Systematized Nomenclature of Medicine—Clinical Terms (SNOMED CT) ^36^, Gene Ontology ^37^, International Classification of Diseases (ICD) ontology ^38^, Dementia-Related Agitation Non-Pharmacological Treatment Ontology (DRANPTO) ^39^, and Dementia-Related Psychotic Symptom Non-Pharmacological Treatment Ontology (DRPSNPTO) ^40^. However, to the best of our knowledge, no ontology currently represents the knowledge in the domain of non-pharmacological treatment of emotional and mood disturbances in dementia. Therefore, this study aims to develop an ontology for this specific domain, named Dementia-Related Emotional And Mood Disturbance Non-Pharmacological Treatment Ontology (DREAMDNPTO).

## Methods

Various methodologies for ontology development have been developed, such as Agile Methodology for Ontology Development ^41^, On-To-Knowledge methodology ^42^, OntoClean methodology ^43^, METHONTOLOGY ^44^, and the NeOn methodology ^45^. We select the NeOn methodology as the baseline methodology for the development of DREAMDNPTO because it provides a well-defined and systematic approach to ontology development that emphasises domain expert involvement, reuse of existing resources, and ontology evaluation ^45^. The NeOn methodology has been used to build many ontologies successfully in the health domain, such as Patient-Practitioner Assistive Communications (PPAC) ontology for type 2 diabetes management ^46^ and the human activity representation ontology to assist old people living independently in smart homes ^47^.

Previously we have applied the NeOn methodology to develop the DRANPTO ontology ^39^ and DRPSNPTO ontology ^40^. DREAMDNPTO was developed using the same method as the DRPSNPTO ontology ^40^ based on the NeOn methodology ^45^. This method contains five main steps – ontology requirement specification, resource reuse, ontology conceptualisation, ontology alignment, and ontology evaluation and refinement. Six experts with experience and knowledge in the target domain participated in developing DREAMDNPTO, including a clinical psychologist, a behavioural neuroscientist, a dementia care specialist, a gerontological nurse, a digital health expert, and a nursing manager with pharmacy training in a long-term care facility (called residential aged care in Australia).

### Step 1: Ontology requirement specification

First, based on a review of the relevant literature, previous research experience in ontology development ^39,40^, and consultations with the domain experts, the basic requirements for DREAMDNPTO were identified. These requirements are: (1) its scope is to include emotional and mood disturbances in dementia, non-pharmacological interventions forelar use in long-term care facilities, and factors that would be required to deliver a person-centred non-pharmacological approach for people with dementia; (2) its intended users are healthcare professionals caring for people with dementia, dementia care researchers, knowledge engineers developing ontological resources related to dementia care, and software engineers building knowledge-driven artificial intelligent systems related to dementia care; (3) its intended use cases are to support the integration of heterogeneous data resources, and to advise healthcare professionals about the appropriate non-pharmacological interventions for emotional and mood disturbances in a person with dementia; and (4) it is able to answer the twelve competency questions (Table 1), which are ontology content-specific requirements represented in the form of natural language questions ^48^. This set of requirements was used as a guide for ontology development.

**Table 1:** Competency questions for DREAMDNPTO to answer.

No. of CQ	Competency questions (CQ)
1	What are emotional and mood disturbances in people with dementia?
2	What causes emotional and mood disturbances in people with dementia living in long-term care facilities?
3	What are the manifestations of emotional and mood disturbances in people with dementia living in long-term care facilities?
4	What are the main activities for managing the emotional and mood disturbances of people with dementia living in long-term care facilities?
5	What tools are used to measure emotional and mood disturbances in people with dementia living in long-term care facilities?
6	What are the care goals for managing the emotional and mood disturbances of people with dementia living in long-term care facilities?
7	What non-pharmacological interventions are used for emotional and mood disturbances of people with dementia living in long-term care facilities?
8	What background information of people with dementia is related to emotional and mood disturbance management in long-term care facilities?
9	What factors affect the implementation of non-pharmacological interventions for emotional and mood disturbances of people with dementia living in long-term care facilities?
10	Who is involved in emotional and mood disturbances management in dementia care in long-term care facilities?
11	What are the effects of emotional and mood disturbances in dementia in long-term care facilities?
12	What communication skills are used to communicate with people with dementia?

### Step 2: Knowledge reuse

We reused three knowledge resources for extracting the knowledge related to non-pharmacological treatment of emotional ad mood disturbance of dementia: relevant existing ontologies, published literature and clinical data of people with dementia living in long-term care facilities. First, we reviewed three ontologies, including the ICD version 11 ^49^, the DRANPTO ontology ^39^, and the DRPSNPTO ontology ^40^, and reused 268 terms in these ontologies (26, 76, and 166 terms, respectively) for developing DREAMDNPTO (Supplementary Material 1). Second, we reviewed relevant articles about the non-pharmacological treatment for emotional and mood disturbances of people with dementia in long-term care facilities that we gathered through a systematic search across three databases (MEDLINE with Full Text, CINAHL Plus with Full Text, and APA PsycINFO) (Supplementary Material 2) and eight authoritative websites, e.g., the Alzheimer’s Association (Supplementary Material 3). Following the Preferred Reporting Items for Systematic Reviews and Meta-Analyses guidelines ^50^, 103 out of 420 articles were selected to build DREAMDNPTO (Supplementary Material 4 for the article selection process and Supplementary Material 5 for article selection criteria). Relevant sentences and terms were extracted and coded using NVivo 12, a software used for qualitative analysis and management of unstructured data ^51^ and analysed by an ontology engineer (ZZ).

Third, after receiving ethical approval from the Human Research Ethics Committee at the University of Wollongong (reference number: 2019/159), we obtained the clinical data of people with dementia living in 40 long-term care facilities in Australia. The dataset included 14,034 de-identified clinical records of 1686 people with dementia that contained information about emotional and mood disturbances. The records include a variety of progress notes, such as weekly care reviews, medical doctor’s notes, and occupational therapist’s notes. These clinical records are written in the free-text form in English (i.e., natural language). We randomly sampled 1000 clinical records of 555 people with dementia to extract the relevant terms and sentences as the resource for developing DREAMDNPTO using Python programming language (version 3.8.8) ^52^ and Pandas (version 1.2.4, a Python library for data manipulation and analysis) ^53^ in the Jupyter Notebook environment (version 6.3.0, and open-source web-based interactive computing environment) ^54^. Sampling concluded when data saturation was reached, i.e., when the number of new terms contributed by 100 new records is less than or equal to 1% of the entire terms of the ontology ^55^. Three new terms were extracted from 100 new records, (3/1258 = 0.24%) < 1%, indicating that data saturation was reached. The use of clinical records in this study was approved by the Institutional Ethical Committee and all experiments were performed in accordance with the University of Wollongong Guidelines on the ethical use of personal information.

### Step 3: Ontology conceptualisation

The Protégé ontology editor (version 5.5.0) ^56^ was applied to create DREAMDNPTO. Classes and object properties were created to represent concepts and their inter-relationships, respectively, derived from the terms in the extracted sentences. In the health domain, it is common for a concept to be represented by several synonymous terms ^57^. When there is more than one candidate term, a preferred term was selected as the label of the class, following the term selection method ^40^. The others were coded as synonyms.

To improve the comprehensibility of DREAMDNPTO and its elements, two types of metadata were created: one is the metadata that refers to the whole ontology, e.g., ontology license information annotation, and the other metadata refers to its elements, e.g., the definition of the class. These metadata enable users to determine whether the entire DREAMDNPTO or some of its elements (e.g., a specific class) suits their needs.

### Step 4: Ontology alignment

To enable efficient use of DREAMDNPTO, its concepts were semantically linked with the concepts of the Unified Medical Language System (UMLS) Metathesaurus (version 2021AA) by manually examining the meaning of each concept ^58^. Once a semantically equivalent concept was identified, its unique conceptual identifier in the UMLS Metathesaurus was coded in the annotation of DREAMDNPTO.

### Step 5: Ontology evaluation and refinement

To check if DREAMDNPTO meets the requirements specified at the beginning of the ontology development and the biomedical ontology criteria, first, six domain experts individually evaluated the accuracy, clarity, completeness, and conciseness ^59^ of the DREAMDNPTO using the adopted semi-structured interview guide for evaluating the DRPSNPTO ontology ^40^. Each expert was asked to answer the evaluation questions about the quality of DREAMDNPTO; then to provide opinions about whether DREAMDNPTO satisfies the specified requirements, which took 33.25 h. DREAMDNPTO was refined following the experts’ comments and suggestions.

Second, DREAMDNPTO was automatically tested using the Pellet reasoner (a computer program that helps in building and evaluating ontologies by checking if the logical relationships between concepts are consistent) ^60^ for its consistency, and the OntOlogy Pitfall Scanner (which is a software tool that helps to detect errors and problems in ontologies) ^61^ for potential ontological pitfalls, e.g., misusing ontology annotations.

Third, to test if DREAMDNPTO could correctly answer the twelve competency questions, we transformed these natural language questions into twelve semantic queries using the SPARQL language (SPARQL, a query language used to retrieve and manipulate data stored in RDF format) ^62^. The query results were validated to test if DREAMDNPTO could meet the specified requirements. In addition, to validate the utility of DREAMDNPTO, the prescribing rule for retrieving the related data (e.g., the effective non-pharmacological interventions) was defined and encoded in the Semantic Web Rule Language (SWRL, a language used to create rules that can be used to make deductions or draw inferences from data stored in RDF format) ^63^. Furthermore, the data of a person with dementia (Resident986) from 60 EHRs were inserted as instances in DREAMDNPTO. These EHRs are related to a variety of care domains and activities, including but not limited to weekly care review, manager review, medical doctor’s notes, specialist’s notes, behaviour management, clinical events, activities of daily living, pastoral care, incidents, case conferences, infections, and lifestyle and social engagements. Then, the rule execution results were manually evaluated to test if the accurate data could be extracted from the DREAMDNPTO using the SWRL rule.

## Results

### The resultant DREAMDNPTO

#### An overview of DREAMDNPTO

**Figure 1:**
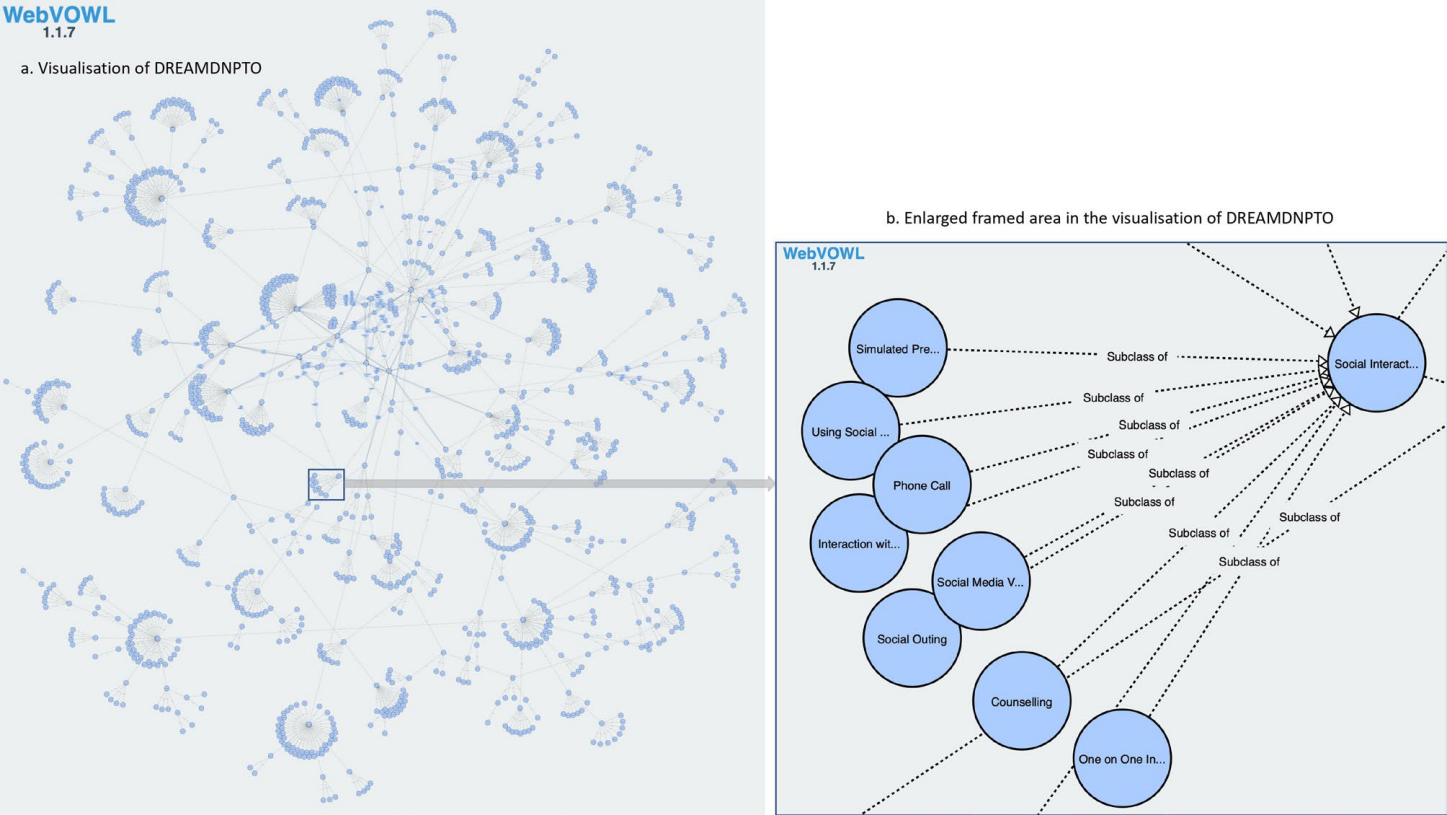
(**a**) Visualisation of DREAMDNPTO in WebVOWL ^66^; (**b**) Enlarged framed area in the visualisation of DREAMDNPTO in WebVOWL. Blue circles depict the classes, dotted lines depict subsumption relationships between the classes, and solid lines depict object properties.

The resultant DREAMDNPTO has 1,258 classes and 70 object properties to represent concepts and their relationships in the non-pharmacological treatment of emotional and mood disturbances in dementia (see Fig. 1). DREAMDNPTO is publicly available at the American National Center for Biomedical Ontology (NCBO) BioPortal ^64^.

DREAMDNPTO has 18 first-level classes (Table 2). Each first-level class has 2 to 350 subclasses organised in two to nine granular levels. The first-level class “Management Activity of Emotional and Mood Disturbance in Dementia”, “Causative Factor of Emotional and Mood Disturbance in Dementia”, and “Background of Care Recipient” are the core of DREAMDNPTO (i.e., the top three first-level classes that have the most subclasses, with 351, 221, and 217 subclasses, respectively, accounting for 62.72% of all subclasses in DREAMDNPTO). “Management Activity of Emotional and Mood Disturbance in Dementia” has the most levels—nine levels, followed by “Causative Factor of Emotional and Mood Disturbance in Dementia” with seven levels, and “Effect of Emotional and Mood Disturbance in Dementia” with six levels.

**Table 2:** Hierarchical distribution of classes in DREAMDNPTO with names of first-level classes and the number of classes at all levels.

Name of First-Level Classes	Number of Classes at Different Levels									Number in total (with %)
	1st	2nd	3rd	4th	5th	6th	7th	8th	9th	
Management Activity of Emotional and Mood Disturbance in Dementia	1	7	31	15	15	135	100	38	9	351 (27.90%)
Background of Care Recipient	1	23	95	82	20	0	0	0	0	221(17.57%)
Causative Factor of Emotional and Mood Disturbance in Dementia	1	3	43	99	55	10	6	0	0	217 (17.25%)
Effect of Emotional and Mood Disturbance in Dementia	1	6	29	10	46	28	0	0	0	120 (9.54%)
Person	1	9	48	17	0	0	0	0	0	75 (5.96%)
Manifestation of Emotional and Mood Disturbance in Dementia	1	14	43	14	1	0	0	0	0	73 (5.80%)
Assessment Tool for Emotional and Mood Disturbance in Dementia	1	48	0	0	0	0	0	0	0	49 (3.90%)
Skill for Communicating with Person with Dementia	1	2	40	0	0	0	0	0	0	43 (3.42%)
Dementia	1	15	8	0	0	0	0	0	0	24 (1.91%)
Time	1	23	0	0	0	0	0	0	0	24 (1.91%)
Feature of Manifestation of Emotional and Mood Disturbance in Dementia	1	3	13	0	0	0	0	0	0	17 (1.35%)
Factor Affecting Implementation of Nonpharmacological Intervention	1	10	2	0	0	0	0	0	0	13 (1.03%)
Care Goal for Emotional and Mood Disturbance in Dementia	1	8	0	0	0	0	0	0	0	9 (0.72%)
Effectiveness Level of Nonpharmacological Intervention	1	2	4	0	0	0	0	0	0	7 (0.56%)
Emotional and Mood Disturbance in Dementia	1	4	0	0	0	0	0	0	0	5 (0.40%)
Stage of Dementia	1	3	0	0	0	0	0	0	0	4 (0.32%)
Management Outcome of Emotional and Mood Disturbance in Dementia	1	2	0	0	0	0	0	0	0	3 (0.24%)
Delivery Mode of Nonpharmacological Intervention	1	2	0	0	0	0	0	0	0	3 (0.24%)

There are two types of metadata for each class—descriptive metadata, including a label which is a human-readable class name, Internationalised Resource Identifier (IRI) short name which is a technical name, definition, abbreviation, synonym, and UMLS mapping information if applicable; and structural metadata, including IRI, direct superclass, direct subclasses, and relationships with other classes if applicable. For example, the class “Reminiscence Therapy” has descriptive metadata including a label—Reminiscence Therapy, an IRI short name—ReminiscenceTherapy, a definition—“using the recall of past events, feelings, and thoughts to facilitate pleasure, quality of life, or adaptation to present circumstances” ^65^, an abbreviation—RT, and UMLS mapping information—a concept unique identifier: C0150321.

The class “Reminiscence Therapy” also has structural metadata including an IRI—http://www.semanticweb.org/zhenyuzhang/ontologies/2021/DREAMDNPTO#ReminiscenceTherapy, a direct superclass—“Therapy for Emotional and Mood Disturbance in Dementia”, and 18 relationships with other classes—“has Target Person” and “is Target Person” with “Person”, “treats” and “is treated by” relationships with “Emotional and Mood Disturbance in Dementia”, “matches” and “is matched by” relationships with “Background of Care Recipient”, “conducts” and “is conducted by” relationships with “Person”, “has time” and “is time of” relationships with “Time”, “has delivery mode” and “is delivery mode of” relationships with “Delivery Mode of Nonpharmacological Intervention”, “has effectiveness level” and “is effectiveness level of” relationships with “Effectiveness Level of Nonpharmacological Intervention”, “has outcome” and “is outcome of” relationships with “Management Outcome of Emotional and Mood Disturbance in Dementia”, “affects implementation of” and “is affected by” relationships with “Factor Affecting Implementation of Nonpharmacological Intervention”. In addition, DREAMDNPTO contains 1,102 synonyms for 532 classes; 601(47.78%) of 1,258 classes were semantically linked to the UMLS Metathesaurus. 

#### Core of DREAMDNPTO: the top three first-level classes

**Figure 2:**
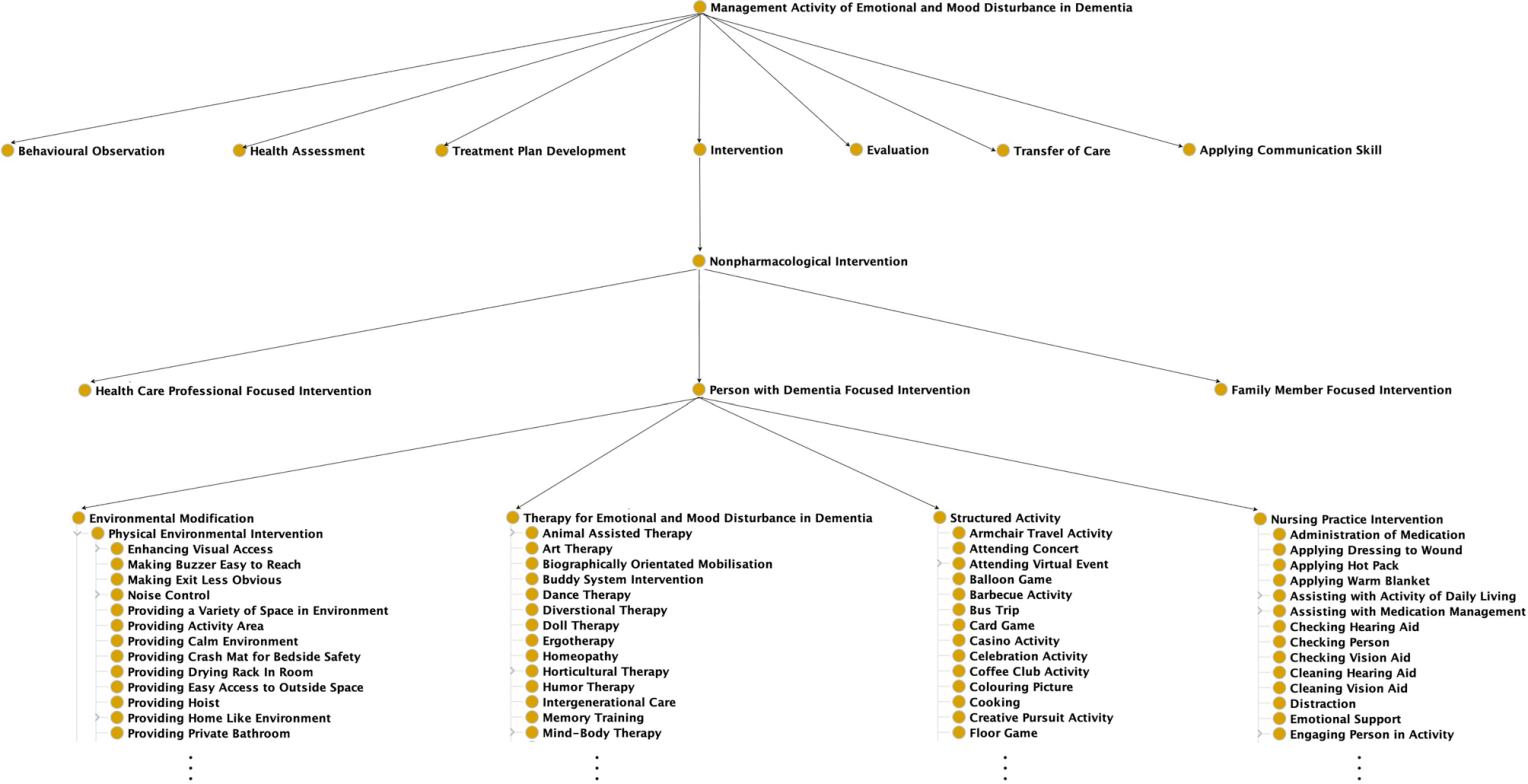
A simplified hierarchical structure of “Management Activity of Emotional and Mood Disturbance in Dementia” with a focus on non-pharmacological interventions.

“Management Activity of Emotional and Mood Disturbance in Dementia” represents the activities conducted for managing emotional and mood disturbances in people with dementia. It has seven direct subclasses, including “Behavioural Observation”, “Health Assessment”, “Treatment Plan Development”, “Intervention”, “Evaluation”, “Transfer of Care”, and “Applying Communication Skill” (Fig. 2). “Intervention” contains “Non-pharmacological Intervention”, representing interventions that do not use drugs. Based on the target group of intervention recipients, these interventions were organised into three categories represented by three classes “Person with Dementia Focused Intervention”, “Health Care Professional Focused Intervention”, and “Family Member Focused Intervention”. Among them, “Person with Dementia Focused Intervention”, representing the non-pharmacological interventions targeting people with dementia, is the class with the most subclasses—282 subclasses. These subclasses were further organised into four lower-level classes “Environmental Modification”, “Therapy for Emotional and Mood Disturbance in Dementia”, “Structured Activity”, and “Nursing Practice Intervention”, containing 38, 95, 56, and 89 subclasses, respectively.

**Figure 3:**
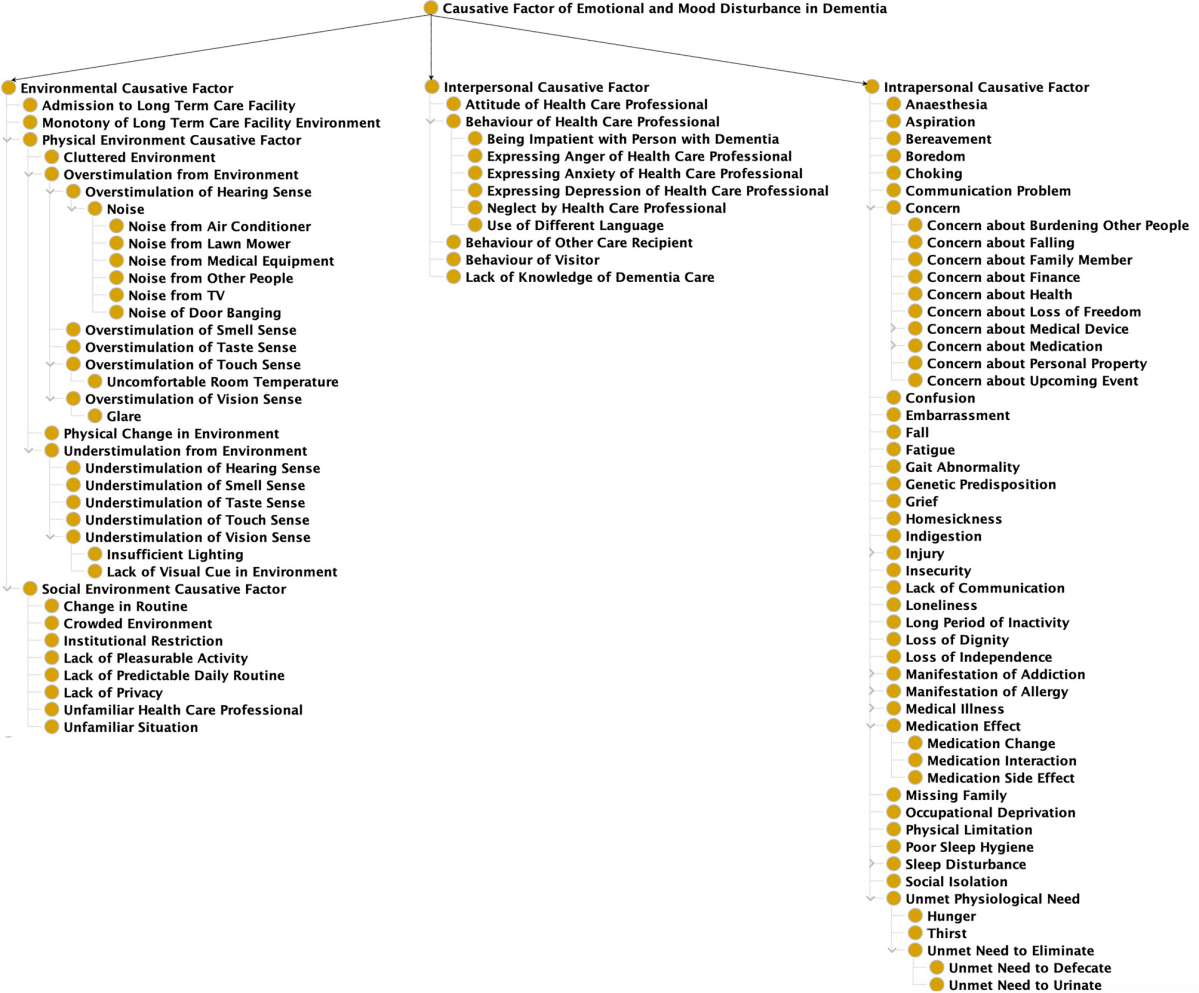
A simplified hierarchical structure of “Causative Factor of Emotional and Mood Disturbance in Dementia”.

“Causative Factor of Emotional and Mood Disturbance in Dementia” is the second top first-level class in DREAMDNPTO, representing the factors that could cause emotional and mood disturbances in people with dementia. It has three direct subclasses—“Environmental Causative Factor”, “Interpersonal Causative Factor”, and “Intrapersonal Causative Factor” (Fig. 3). Each has 37, 11, and 165 lower-level subclasses, respectively.

**Figure 4:**
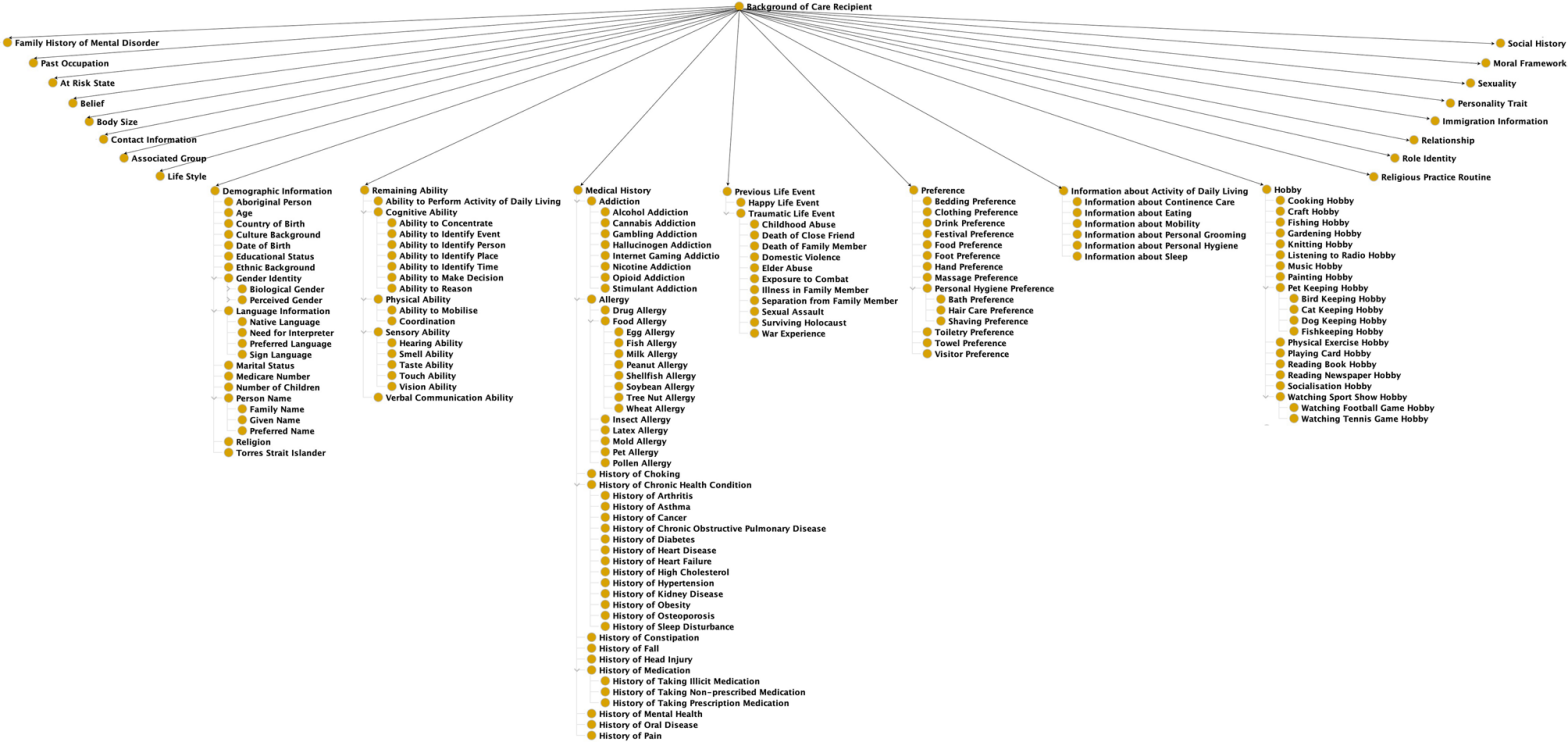
A simplified hierarchical structure of “Background of Care Recipient”.

“Background of Care Recipient” is the third top first-level class in DREAMDNPTO, representing the concepts of the background information about a care recipient – a person with dementia. It has 23 direct subclasses, e.g., “Previous Life Event”, “Hobby”, and “Medical History” (Fig. 4).

### Results of evaluating DREAMDNPTO

#### Results of ontology evaluation against biomedical ontology criteria

DREAMDNPTO was evaluated against the biomedical ontology criteria, including consistency, clarity, completeness, conciseness, and accuracy ^59^. The reasoner Pellet's evaluation results confirmed the logical consistency of DREAMDNPTO. Regarding clarity, the domain experts identified that labels or definitions of 116 classes were difficult to understand or ambiguous, and they recommended new terms and definitions to replace them. For example, “Review of Laboratory Test” was replaced by “Review of Pathology Test”.

Regarding completeness, the domain experts suggested adding 178 new classes to DREAMDNPTO representing new concepts, e.g., “Aquatic Exercise”. They also suggested adding nine synonyms to the related classes of DREAMDNPTO. For example, the term “Expressive Music Therapy” was added as a synonym for “Active Music Therapy”.

Regarding conciseness, 65 classes were excluded from DREAMDNPTO because the domain experts identified them as either out of the specified ontology scope or semantically duplicated with other classes. For example, “Analgesia” was excluded because it relates to pharmacological interventions, hence falling outside of the scope.

Regarding accuracy, 200 issues were discovered (150 by domain experts and 50 by the OntOlogy Pitfall Scanner). The issues discovered by domain experts were solved with their recommendations; those by the OntOlogy Pitfall Scanner were manually checked and confirmed as the correct semantic representation. In conclusion, DREAMDNPTO meets the biomedical ontology criteria.

#### Results of ontology evaluation against specified requirements

Domain experts verified that DREAMDNPTO meets the first two of the four requirements listed in 2.1. The data inferred from the retrieval results according to the SWRL rule were the same as expected, validating the third requirement—the utility of DREAMDNPTO (Supplementary Material 6). Regarding the fourth requirement, the SPARQL results show that DREAMDNPTO can correctly answer competency questions. Taking competency question No.6 as an example (Supplementary Material 7), the retrieved data were the eight care goals for managing emotional and mood disturbances of people with dementia in long-term care facilities in DREAMDNPTO presented in alphabetical order, encompassing both IRI short names and corresponding labels. The retrieved data is consistent with the original concepts.

## Discussion

To the best of our knowledge, this study develops the first ontology—DREAMDNPTO—a novel semantic representation of knowledge in the non-pharmacological treatment for emotional and mood disturbances of people with dementia living in long-term care facilities. It aligns with the World Health Organization's priorities of assisting people with dementia and their caregivers through the use of technology ^67^.

**Figure 5:**
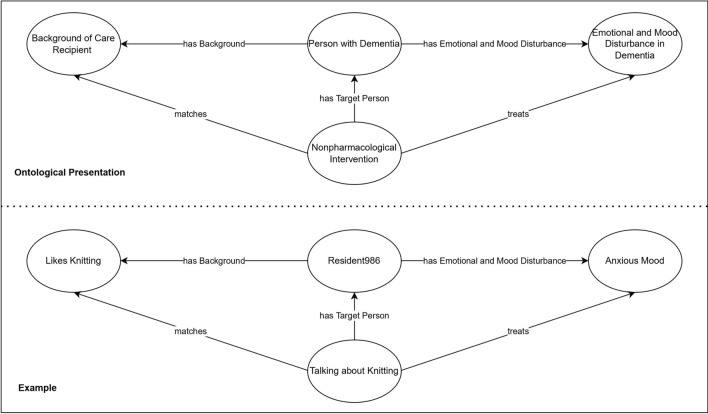
Data of Resident986 as individuals of DREAMDNPTO.

DREAMDNPTO comprehensively contains the 1,258 concepts of non-pharmacological management for emotional and mood disturbances in dementia. These concepts were semantically arranged to adhere to the guiding principle of dementia care—person-centred care, emphasising the importance of understanding the person and placing them in the centre of care ^68^. DREAMDNPTO contains 217 concepts to understand the background of a person with dementia (e.g., hobbies and traumatic life events). These concepts serve as the foundation of person-centred care to people with dementia who experience emotional and mood disturbances. Additionally, the class “Person with Dementia” is linked to these background concepts through the relationship “has Background”, to emotional and mood disturbances in dementia through “has Emotional and Mood Disturbance in Dementia”, and to non-pharmacological interventions through the relationship “has Target Person” (Fig. 5). This arrangement of concepts ensures that the non-pharmacological interventions place the person in the centre of care and treat the person’s specific emotional and mood disturbances according to their personal background. It enables DREAMDNPTO to capture detailed personal information for providing effective person-centred non-pharmacological interventions to the emotional and mood disturbances of this person. For example, Resident986 has a hobby of knitting. Thus, talking about knitting with her is an effective person-centred intervention for managing her anxious mood.

In addition, DREAMDNPTO includes new concepts not included in other ontologies, such as music therapeutic caregiving—care staff singing during assisting a person with dementia in moving from one position to another ^69^, and scheduled reassurance therapy—“brief 1 min interactions every 30 min consisting of positive social interactions where no nursing/care tasks are performed” ^70^. Including these new concepts not only ensures the coverage of DREAMDNPTO but also increases the conceptual richness in terms of the overall semantic representation of biomedical knowledge.

DREAMDNPTO’s utility is demonstrated by successfully retrieving non-pharmacological interventions for a person with dementia-related emotional and mood disturbances from various EHRs. This implies that the clinical decision-support systems based on DREAMDNPTO as the knowledge model can assist healthcare professionals in extracting information about relevant person-centred non-pharmacological interventions, effectively resolving the challenge of information retrieval in current clinical practice. For example, Resident986 has dementia and is often anxious because of concerns about her husband’s poor health. Six effective non-pharmacological interventions were inferred, including distracting the resident by talking about knitting, pet therapy, one-to-one intervention, reassuring the resident, reorienting the resident to time, and reorienting the resident to place. According to the clinical care records of this person, these interventions are effective in managing her anxious mood because they are tailored to her hobbies (knitting and pets), unique needs (assurance for her husband’s health), and health conditions (cognitive impairment and psychosis). The approach to delivering these interventions to assist healthcare professionals could replace the “trial-and-error” approach and enhance the quality of care for people with dementia in long-term care facilities. Moreover, it could relieve the burden on healthcare professionals in managing emotional and mood disturbances in dementia.

The successful retrieval of an individual’s non-pharmacological interventions from various EHRs also demonstrates that DREAMDNPTO can support the integration of heterogeneous resources. Because integrating information is the pre-step for information processing and reasoning. For example, to retrieve the effective non-pharmacological interventions for Resident986, 60 EHRs were manually coded as the instances of DREAMDNPTO. These encoded data were automatically linked together by DREAMDNPTO, forming a holistic picture of Resident986. It allows users to efficiently access the information they need and solves the challenge of finding relevant information in the large amount of unstructured EHRs.

Manually coding EHRs for DREAMDNPTO evaluation was time-consuming and labour-intensive. Natural language processing and machine learning technologies can automatically extract related data from EHRs ^71,72^. These technologies will allow DREAMDNPTO to semantically link a complex range of data related to the management of emotional and mood disturbances in dementia at a scale that has been lacking. A large amount of data that linked to DREAMDNPTO will provide a solid foundation for the computerised algorithm to conduct detailed verbatim analysis, eventually generating meaningful insights about emotional and mood disturbances in dementia.

Notably, the current DREAMDNPTO focuses on the non-pharmacological interventions for people with dementia living in long-term care settings rather than home care settings, where resources (e.g., EHRs) are limited. We may subsequently update DREAMDNPTO to make it applicable to dementia care in home care settings.

## Conclusions

This study developed DREAMDNPTO, which represents the knowledge of non-pharmacological treatment for emotional and mood disturbances of people with dementia living in long-term care facilities. It satisfies the requirements and the standards for biomedical ontology. DREAMDNPTO paves the way for developing ontology-based applications for managing emotional and mood disturbances in dementia, such as recommending appropriate interventions, answering related questions, and integrating heterogeneous resources.

### Supplementary Information

Supplementary Information.

## Data Availability

The developed DREAMDNPTO ontology is publicly available at the NCBO BioPortal https://bioportal.bioontology.org/ontologies/DREAMDNPTO.
