# Supplementary material for: Developing an ontology of non-pharmacological treatment for emotional and mood disturbances in dementia
Source: Sci Rep. 2024 Jan 22;14:1937. doi: 10.1038/s41598-023-46226-5 (PMC10803746; doi:10.1038/s41598-023-46226-5)
Supplement: Supplementary file 1 — Supplementary Information. [file 41598_2023_46226_MOESM1_ESM.pdf]

Supplementary Material

Developing an Ontology of Non-Pharmacological Treatment for Emotional and Mood Disturbances in Dementia

Zhenyu Zhang[1](0000-0003-1853-4978), Ping Yu[1,2,*](0000-0002-7910-9396), Mengyang Yin[1,3](0000-0002-0212-4598), Hui Chen Chang[2,4](0000-0002-8305-0585), Susan J Thomas[2,5](0000-0003-3609-4443), Wenxi Wei[4](0000-0001-7641-948X), Ting Song[1,2](0000-0001-5858-6495), Chao Deng[2,6](0000-0003-1147-5741)

[1]Centre for Digital Transformation, School of Computing and Information Technology, University of Wollongong, Wollongong, Australia;

[2]Illawarra Health and Medical Research Institute, University of Wollongong, Wollongong, Australia;

[3]Systems and Reporting Residential Care, Catholic Healthcare Ltd, Wollongong, Australia;

[4]School of Nursing, University of Wollongong, Wollongong, Australia;

[5]Graduate School of Medicine, University of Wollongong, Wollongong, Australia;

[6]School of Medical, Indigenous and Health Sciences, University of Wollongong, Wollongong, Australia

*Corresponding Author: Ping Yu, Northfield Ave, School of Computing and Information Technology, University of Wollongong, Wollongong, NSW 2522, Australia. Email: ping@uow.edu.au

Supplementary Material 1

Summary of the terms reused from the ICD version 11, DRANPTO and DRPSNPTO ontologies for developing DREAMDNPTO

Ontologies	Number of Reused Terms	Examples of Reused Terms
ICD-11	• 24 terms representing types of dementia	• Dementia due to Lewy Body Disease
	• 2 terms representing emotional and mood disturbances in dementia	• Apathy in Dementia
DRANPTO	• 76 terms representing agitated behaviours in dementia	• Punching
DRPSNPTO	• 106 terms representing background information of people with dementia	• Medical History
	• 3 terms representing factors that affect the implementation of non-pharmacological interventions in the long-term care facilities	• Organizational Culture
	• 33 terms representing people involved in the care of people with dementia in the long-term care facilities	• Registered Nurse
	• 6 terms representing communication skills for communicating with people with dementia	• Active Listening
	• 18 terms representing time in the long-term care facilities	• Breakfast Time

Supplementary Material 2

Search terms for identifying relevant articles about non-pharmacological treatment for emotional and mood disturbances in dementia: All three databases and eight authoritative websites were searched with the following search terms: ("dementia") AND ("mood disorder" OR "mood symptom" OR "apathy" OR "anxiety" OR "depression" OR "euphoria") AND ("nonpharmacological interventions" OR "nonpharmacological treatment" OR "nonpharmacological therapy").

Supplementary Material 3

Eight authoritative websites about dementia:

- Dementia Action Alliance
- Dementia Australia
- StatPearls
- The Alzheimer's Association
- The International Psychogeriatric Association
- The American Psychiatric Association
- The British Geriatric Society
- The American Geriatric Society

Supplementary Material 4

Article selection process

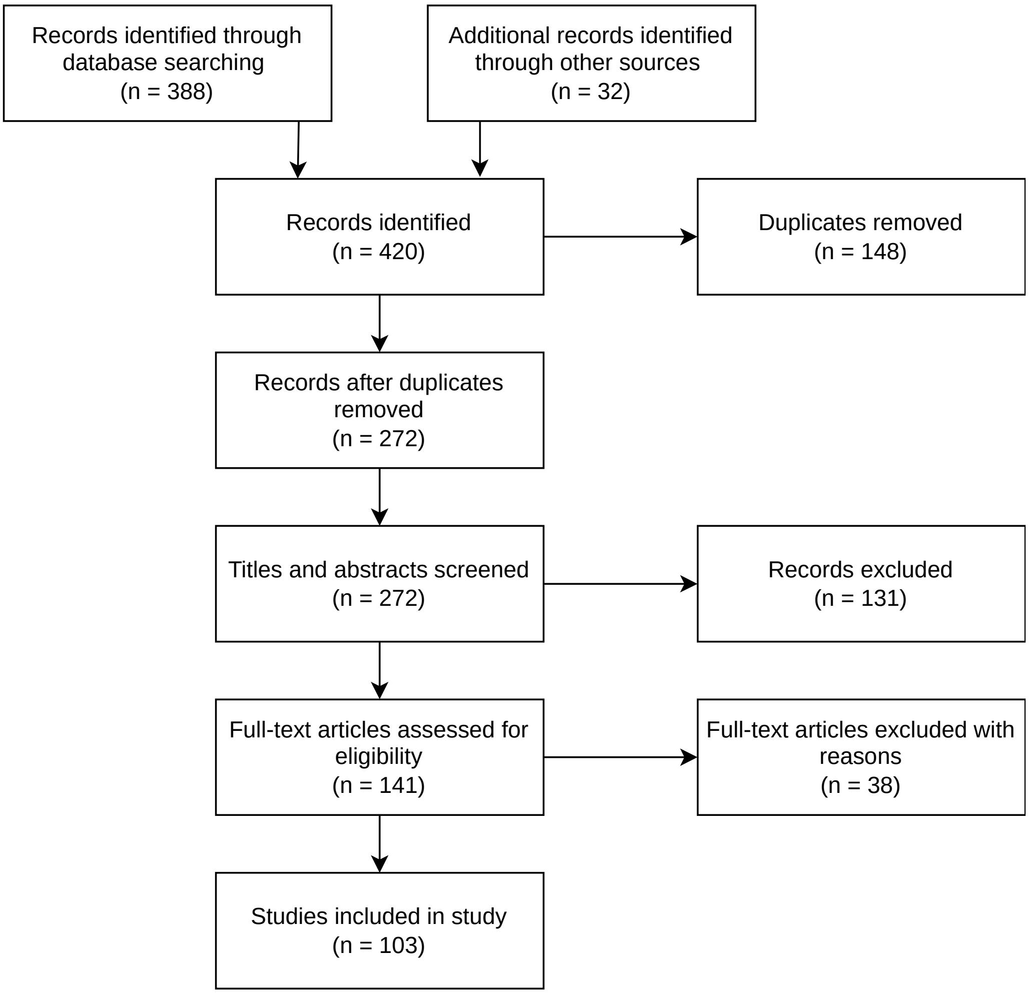

Supplementary Material 5

Article selection criteria

Inclusion Criteria

- Articles written in English
- Articles published from database inception onward to 17th February 2021
- Articles published in peer-reviewed scientific journals or authoritative websites
- Studies conducted on the population of older adults with dementia
- Articles include information about non-pharmacological treatment for emotional and mood disturbances including apathy, anxiety, depression, and euphoria for people with dementia (of any type or severity) living in long-term care facilities
- Types of studies include case studies, descriptive studies, systematic reviews, meta-analyses, and randomised controlled trials of nonpharmacological treatment including all study designs, as well as practical guidelines for emotional and mood disturbances for people with dementia living in long-term care facilities

Exclusion Criteria

- Studies conducted in a population other than older adults with dementia
- Articles about pharmacological interventions for emotional and mood disturbances in people with dementia
- Articles about multicomponent interventions with a component of pharmacological interventions
- Studies focused on an overall outcome of BPSD but not specifically reducing emotional and mood disturbances in people with dementia
- Articles about electroconvulsive therapy
- Studies conducted in the home care environment, hospital environment, and community day care centre
- Articles only contain the study protocol without outcome
- Conference abstract, poster abstract, and editorial material

Supplementary Material 6

The defined SWRL rule and results

SWRL Rule
PersonWithDementia(?resident)^<br>hasEmotionalAndMoodDisturbance(?resident, ?emotional_and_mood_disturbance)^<br>hasManifestation(?emotional_and_mood_disturbance, ?manifestation)^<br>causes(?causative_factor, ?emotional_and_mood_disturbance)^<br>treats(?nonpharmacological_intervention, ?emotional_and_mood_disturbance)<br>hasEffectivenessLevel(?nonpharmacological_intervention, InterventionIsEffective)<br>-> sqwrl:select(?resident, ?emotional_and_mood_disturbance, ?manifestation, ?causative_factor, ?nonpharmacological_intervention)

Results

resident	emotional_and_mood_disturbance	manifestation	causative_factor	nonpharmacological_intervention
DREAMDNPTO:Resident986	DREAMDNPTO:BeingAnxious	DREAMDNPTO:BeingWorried	DREAMDNPTO:BadHealthOfResidentHusband	DREAMDNPTO:TalkingAboutKnitting
DREAMDNPTO:Resident986	DREAMDNPTO:BeingAnxious	DREAMDNPTO:BeingWorried	DREAMDNPTO:BadHealthOfResidentHusband	DREAMDNPTO:PetTherapy
DREAMDNPTO:Resident986	DREAMDNPTO:BeingAnxious	DREAMDNPTO:BeingWorried	DREAMDNPTO:BadHealthOfResidentHusband	DREAMDNPTO:OneToOneIntervention
DREAMDNPTO:Resident986	DREAMDNPTO:BeingAnxious	DREAMDNPTO:BeingWorried	DREAMDNPTO:BadHealthOfResidentHusband	DREAMDNPTO:ReorientingResidentToTime
DREAMDNPTO:Resident986	DREAMDNPTO:BeingAnxious	DREAMDNPTO:BeingWorried	DREAMDNPTO:BadHealthOfResidentHusband	DREAMDNPTO:GivingReassurance
DREAMDNPTO:Resident986	DREAMDNPTO:BeingAnxious	DREAMDNPTO:BeingWorried	DREAMDNPTO:BadHealthOfResidentHusband	DREAMDNPTO:ReorientingResidentToPlace
DREAMDNPTO:Resident986	DREAMDNPTO:BeingDepressed	DREAMDNPTO:BeingTeary	DREAMDNPTO:BelievingThatSheIsGoingToJail	DREAMDNPTO:PlayingCards
DREAMDNPTO:Resident986	DREAMDNPTO:BeingDepressed	DREAMDNPTO:BeingTeary	DREAMDNPTO:BelievingThatSheIsGoingToJail	DREAMDNPTO:GivingReassurance
DREAMDNPTO:Resident986	DREAMDNPTO:BeingDepressed	DREAMDNPTO:BeingTeary	DREAMDNPTO:BelievingThatSheIsGoingToJail	DREAMDNPTO:HappyHour
DREAMDNPTO:Resident986	DREAMDNPTO:BeingDepressed	DREAMDNPTO:BeingTeary	DREAMDNPTO:BelievingThatSheIsGoingToJail	DREAMDNPTO:Movie
DREAMDNPTO:Resident986	DREAMDNPTO:BeingDepressed	DREAMDNPTO:BeingTeary	DREAMDNPTO:BelievingThatSheIsGoingToJail	DREAMDNPTO:OneToOneIntervention
DREAMDNPTO:Resident986	DREAMDNPTO:BeingDepressed	DREAMDNPTO:BeingTeary	DREAMDNPTO:BelievingThatSheIsGoingToJail	DREAMDNPTO:Bingo
DREAMDNPTO:Resident986	DREAMDNPTO:BeingDepressed	DREAMDNPTO:BeingTeary	DREAMDNPTO:BelievingThatSheIsGoingToJail	DREAMDNPTO:PastoralCareVisit

Supplementary Material 7

SPARQL query and query results for competency question No.1 “What are emotional and mood disturbances in people with dementia?”

SPARQL query:

```
PREFIX rdf: <http://www.w3.org/1999/02/22-rdf-syntax-ns#>
PREFIX owl: <http://www.w3.org/2002/07/owl#>
PREFIX rdfs: <http://www.w3.org/2000/01/rdf-schema#>
PREFIX xsd: <http://www.w3.org/2001/XMLSchema#>
PREFIX :<http://www.semanticweb.org/zhenyuzhang/ontologies/2021/DREAMDNPTO#>
SELECT ?internationalised_resource_identifier_short_name_of_emotional_and_mood_disturbance
?label_of_emotional_and_mood_disturbance
        WHERE { ?internationalised_resource_identifier_short_name_of_emotional_and_mood_disturbance rdfs:subClassOf :
EmotionalAndMoodDisturbanceInDementia;
rdfs:label ?label_of_emotional_and_mood_disturbance. } ORDER BY ?label_of_emotional_and_mood_disturbance
```

internationalised_resource_identifier_short_name_of_emotional_and_mood_disturbance	label_of_emotional_and_mood_disturbance
AnxietyInDementia	"Anxiety in Dementia"@en
ApathyInDementia	"Apathy in Dementia"@en
DepressionInDementia	"Depression in Dementia"@en
EuphoriaInDementia	"Euphoria in Dementia"@en

SPARQL query and query results for competency question No.2 “What causes emotional and mood disturbances in people with dementia living in long-term care facilities?”

SPARQL query:

```
PREFIX rdf: <http://www.w3.org/1999/02/22-rdf-syntax-ns#>
PREFIX owl: <http://www.w3.org/2002/07/owl#>
PREFIX rdfs: <http://www.w3.org/2000/01/rdf-schema#>
PREFIX xsd: <http://www.w3.org/2001/XMLSchema#>
PREFIX :<http://www.semanticweb.org/zhenyuzhang/ontologies/2021/DREAMDNPTO#>
SELECT ?internationalised_resource_identifier_short_name_of_causative_factor ?label_of_causative_factor
            WHERE { ?internationalised_resource_identifier_short_name_of_causative_factor rdfs:subClassOf*
:CausativeFactorOfEmotionalAndMoodDisturbanceInDementia;
rdfs:label ?label_of_causative_factor. } ORDER BY ?label_of_causative_factor
```

internationalised_resource_identifier_short_name_of_causative_factor	label_of_causative_factor
ActivitiesOfDailyLivingImpairment	"Activities of Daily Living Impairment"@en
AcuteMyocardialInfarction	"Acute Myocardial Infarction"@en
AdmissionToLongTermCareFacility	"Admission to Long Term Care Facility"@en
Amnesia	"Amnesia"@en
Anaemia	"Anaemia"@en
Anaesthesia	"Anaesthesia"@en
AnxietyDisorder	"Anxiety Disorder"@en
Aphasia	"Aphasia"@en
Arthritis	"Arthritis"@en
Aspiration	"Aspiration"@en
AttitudeOfHealthCareProfessional	"Attitude of Health Care Professional"@en
AuditoryImpairment	"Auditory Impairment"@en
BehaviourOfHealthCareProfessional	"Behaviour of Health Care Professional"@en
BehaviourOfOtherCareRecipient	"Behaviour of Other Care Recipient"@en
BehaviourOfVisitor	"Behaviour of Visitor"@en
BeingImpatientWithPersonWithDementia	"Being Impatient with Person with Dementia"@en
Bereavement	"Bereavement"@en
BipolarDisorder	"Bipolar Disorder"@en
Boredom	"Boredom"@en
CausativeFactorOfEmotionalAndMoodDisturbanceInDementia	"Causative Factor of Emotional and Mood Disturbance in Dementia"@en
ChangeInRoutine	"Change in Routine"@en
Choking	"Choking"@en
ClutteredEnvironment	"Cluttered Environment"@en
CognitiveImpairment	"Cognitive Impairment"@en
CommunicationProblem	"Communication Problem"@en
Concern	"Concern"@en
ConcernAboutBurdeningOtherPeople	"Concern about Burdening Other People"@en
ConcernAboutFalling	"Concern about Falling"@en
ConcernAboutFamilyMember	"Concern about Family Member"@en
ConcernAboutFinance	"Concern about Finance"@en
ConcernAboutHealth	"Concern about Health"@en
ConcernAboutIndwellingBladderCatheter	"Concern about Indwelling Bladder Catheter"@en
ConcernAboutInsertedSuprapbicCatheter	"Concern about Inserted Suprapbic Catheter"@en
ConcernAboutLiftingAssistiveDevice	"Concern about Lifting Assistive Device"@en
ConcernAboutLossOfFreedom	"Concern about Loss of Freedom"@en
ConcernAboutMedicalDevice	"Concern about Medical Device"@en
ConcernAboutMedication	"Concern about Medication"@en
ConcernAboutMedicationShortage	"Concern about Medication Shortage"@en
ConcernAboutMedicationSideEffect	"Concern about Medication Side Effect"@en
ConcernAboutPersonalProperty	"Concern about Personal Property"@en
ConcernAboutTakingMedicationIncorrectly	"Concern about Taking Medication Incorrectly"@en
ConcernAboutUpcomingEvent	"Concern about Upcoming Event"@en
ConcernAboutWheeledWalker	"Concern about Wheeled Walker"@en
Confusion	"Confusion"@en
Constipation	"Constipation"@en
CrowdedEnvironment	"Crowded Environment"@en
DaytimeSleepiness	"Daytime Sleepiness"@en
DegenerativePolyarthritis	"Degenerative Polyarthritis"@en
Dehydration	"Dehydration"@en
Delirium	"Delirium"@en
Delusion	"Delusion"@en
DentalDisease	"Dental Disease"@en
DepressiveDisorder	"Depressive Disorder"@en

DiabetesMellitus	"Diabetes Mellitus"@en
Diarrhoea	"Diarrhoea"@en
Discomfort	"Discomfort"@en
DiscomfortFromIllFittingDenture	"Discomfort from Ill-Fitting Denture"@en
DiscomfortFromIllFittingShoe	"Discomfort from Ill-Fitting Shoe"@en
Disorientation	"Disorientation"@en
Dysarthria	"Dysarthria"@en
Dysphasia	"Dysphasia"@en
Dyspnea	"Dyspnea"@en
Eczema	"Eczema"@en
ElectrolyteDisturbance	"Electrolyte Disturbance"@en
Embarrassment	"Embarrassment"@en
EndocrineImbalance	"Endocrine Imbalance"@en
EnvironmentalCausativeFactor	"Environmental Causative Factor"@en
Epilepsy	"Epilepsy"@en
ExecutiveDysfunction	"Executive Dysfunction"@en
ExpressingAngerOfHealthCareProfessional	"Expressing Anger of Health Care Professional"@en
ExpressingAnxietyOfHealthCareProfessional	"Expressing Anxiety of Health Care Professional"@en
ExpressingDepressionOfHealthCareProfessional	"Expressing Depression of Health Care Professional"@en
Fall	"Fall"@en
Fatigue	"Fatigue"@en
FecalIncontinence	"Fecal Incontinence"@en
Fever	"Fever"@en
Fracture	"Fracture"@en
GaitAbnormality	"Gait Abnormality"@en
GastroesophagealRefluxDisease	"Gastroesophageal Reflux Disease"@en
GeneticPredisposition	"Genetic Predisposition"@en
Glare	"Glare"@en
Grief	"Grief"@en
GustatoryImpairment	"Gustatory Impairment"@en
HaematologicalDisease	"Haematological Disease"@en
Haemorrhage	"Haemorrhage"@en
Hallucination	"Hallucination"@en
Homesickness	"Homesickness"@en
Hunger	"Hunger"@en
Hypercholesterolemia	"Hypercholesterolemia"@en
Hypocholesterolemia	"Hypocholesterolemia"@en
Incontinence	"Incontinence"@en
Indigestion	"Indigestion"@en
Infection	"Infection"@en
Injury	"Injury"@en
Insecurity	"Insecurity"@en
Insomnia	"Insomnia"@en
InstitutionalRestriction	"Institutional Restriction"@en
InsufficientLighting	"Insufficient Lighting"@en
InterpersonalCausativeFactor	"Interpersonal Causative Factor"@en
IntrapersonalCausativeFactor	"Intrapersonal Causative Factor"@en
ItchOfSkin	"Itch of Skin"@en
LackOfCommunication	"Lack of Communication"@en
LackOfKnowledgeOfDementiaCare	"Lack of Knowledge of Dementia Care"@en
LackOfPleasurableActivity	"Lack of Pleasurable Activity"@en
LackOfPredictableDailyRoutine	"Lack of Predictable Daily Routine"@en
LackOfPrivacy	"Lack of Privacy"@en
LackOfSpatialAwareness	"Lack of Spatial Awareness"@en
LackOfVisualCueInEnvironment	"Lack of Visual Cue in Environment"@en
Loneliness	"Loneliness"@en
LongPeriodOfInactivity	"Long Period of Inactivity"@en
LossOfDignity	"Loss of Dignity"@en
LossOfIndependence	"Loss of Independence"@en
Malnutrition	"Malnutrition"@en
ManifestationOfAddiction	"Manifestation of Addiction"@en
ManifestationOfAlcoholAddiction	"Manifestation of Alcohol Addiction"@en
ManifestationOfAllergy	"Manifestation of Allergy"@en
ManifestationOfCannabisAddiction	"Manifestation of Cannabis Addiction"@en
ManifestationOfDrugAllergy	"Manifestation of Drug Allergy"@en
ManifestationOfEggAllergy	"Manifestation of Egg Allergy"@en
ManifestationOfFishAllergy	"Manifestation of Fish Allergy"@en

ManifestationOfFoodAllergy	"Manifestation of Food Allergy"@en
ManifestationOfGamblingAddiction	"Manifestation of Gambling Addiction"@en
ManifestationOfHallucinogenAddiction	"Manifestation of Hallucinogen Addiction"@en
ManifestationOfInsectAllergy	"Manifestation of Insect Allergy"@en
ManifestationOfInternetGamingAddiction	"Manifestation of Internet Gaming Addiction"@en
ManifestationOfMilkAllergy	"Manifestation of Milk Allergy"@en
ManifestationOfMoldAllergy	"Manifestation of Mould Allergy"@en
ManifestationOfNicotineAddiction	"Manifestation of Nicotine Addiction"@en
ManifestationOfOpioidAddiction	"Manifestation of Opioid Addiction"@en
ManifestationOfPeanutAllergy	"Manifestation of Peanut Allergy"@en
ManifestationOfPetAllergy	"Manifestation of Pet Allergy"@en
ManifestationOfPollenAllergy	"Manifestation of Pollen Allergy"@en
ManifestationOfShellfishAllergy	"Manifestation of Shellfish Allergy"@en
ManifestationOfSoybeanAllergy	"Manifestation of Soybean Allergy"@en
ManifestationOfStimulantAddiction	"Manifestation of Stimulant Addiction"@en
ManifestationOfTreeNutAllergy	"Manifestation of Tree Nut Allergy"@en
ManifestationOfWheatAllergy	"Manifestation of Wheat Allergy"@en
ManifestationOfLatexAllergy	"Manifestion of Latex Allergy"@en
MedicalIllness	"Medical Illness"@en
MedicationChange	"Medication Change"@en
MedicationEffect	"Medication Effect"@en
MedicationInteraction	"Medication Interaction"@en
MedicationSideEffect	"Medication Side Effect"@en
MemoryImpairment	"Memory Impairment"@en
MentalDisorder	"Mental Disorder"@en
MetabolicDisease	"Metabolic Disease"@en
MissingFamily	"Missing Family"@en
MonotonyOfLongTermCareFacilityEnvironment	"Monotony of Long Term Care Facility Environment"@en
Nausea	"Nausea"@en
NeglectByHealthCareProfessional	"Neglect by Health Care Professional"@en
Neuropathy	"Neuropathy"@en
NightmareDisorder	"Nightmare Disorder"@en
Noise	"Noise"@en
NoiseFromAirConditioner	"Noise from Air Conditioner"@en
NoiseFromLawnMower	"Noise from Lawn Mower"@en
NoiseFromMedicalEquipment	"Noise from Medical Equipment"@en
NoiseFromOtherPeople	"Noise from Other People"@en
NoiseFromTV	"Noise from TV"@en
NoiseOfDoorBanging	"Noise of Door Banging"@en
ObstructiveSleepApnea	"Obstructive Sleep Apnea"@en
OccupationalDeprivation	"Occupational Deprivation"@en
Oedema	"Oedema"@en
OlfactoryImpairment	"Olfactory Impairment"@en
OralDisease	"Oral Disease"@en
OverstimulationFromEnvironment	"Overstimulation from Environment"@en
OverstimulationOfHearingSense	"Overstimulation of Hearing Sense"@en
OverstimulationOfSmellSense	"Overstimulation of Smell Sense"@en
OverstimulationOfTasteSense	"Overstimulation of Taste Sense"@en
OverstimulationOfTouchSense	"Overstimulation of Touch Sense"@en
OverstimulationOfVisionSense	"Overstimulation of Vision Sense"@en
Pain	"Pain"@en
Palpitation	"Palpitation"@en
PanicDisorder	"Panic Disorder"@en
PeripheralVascularDisease	"Peripheral Vascular Disease"@en
PhysicalChangeInEnvironment	"Physical Change in Environment"@en
PhysicalEnvironmentCausativeFactor	"Physical Environment Causative Factor"@en
PhysicalLimitation	"Physical Limitation"@en
PoorSleepHygiene	"Poor Sleep Hygiene"@en
PostTraumaticStressDisorder	"Post Traumatic Stress Disorder"@en
PressureInjury	"Pressure Injury"@en
Prosopagnosia	"Prosopagnosia"@en
PsychiatricComorbidity	"Psychiatric Comorbidity"@en
Psychosis	"Psychosis"@en

Rash	"Rash"@en
Schizophrenia	"Schizophrenia"@en
SensoryImpairment	"Sensory Impairment"@en
SkinIrritation	"Skin Irritation"@en
SkinProblem	"Skin Problem"@en
SleepDeprivation	"Sleep Deprivation"@en
SleepDisturbance	"Sleep Disturbance"@en
SocialEnvironmentCausativeFactor	"Social Environment Causative Factor"@en
SocialIsolation	"Social Isolation"@en
Spasm	"Spasm"@en
Stroke	"Stroke"@en
SubduralHematoma	"Subdural Hematoma"@en
Sundowning	"Sundowning"@en
SwallowingProblem	"Swallowing Problem"@en
TactileImpairment	"Tactile Impairment"@en
Thirst	"Thirst"@en
Tremor	"Tremor"@en
UncomfortableRoomTemperature	"Uncomfortable Room Temperature"@en
UnderstimulationFromEnvironment	"Understimulation from Environment"@en
UnderstimulationOfHearingSense	"Understimulation of Hearing Sense"@en
UnderstimulationOfSmellSense	"Understimulation of Smell Sense"@en
UnderstimulationOfTasteSense	"Understimulation of Taste Sense"@en
UnderstimulationOfTouchSense	"Understimulation of Touch Sense"@en
UnderstimulationOfVisionSense	"Understimulation of Vision Sense"@en
UnfamiliarHealthCareProfessional	"Unfamiliar Health Care Professional"@en
UnfamiliarSituation	"Unfamiliar Situation"@en
UnmetNeedToDefecate	"Unmet Need to Defecate"@en
UnmetNeedToEliminate	"Unmet Need to Eliminate"@en
UnmetNeedToUrinate	"Unmet Need to Urinate"@en
UnmetPhysiologicalNeed	"Unmet Physiological Need"@en
UrinaryIncontinence	"Urinary Incontinence"@en
UrinaryRetention	"Urinary Retention"@en
UseOfDifferentLanguage	"Use of Different Language"@en
VisualImpairment	"Visual Impairment"@en

SPARQL query and query results for competency question No.3 “What are the manifestations of emotional and mood disturbances in people with dementia living in long-term care facilities?”

SPARQL query:

```
PREFIX rdf: <http://www.w3.org/1999/02/22-rdf-syntax-ns#>
PREFIX owl: <http://www.w3.org/2002/07/owl#>
PREFIX rdfs: <http://www.w3.org/2000/01/rdf-schema#>
PREFIX xsd: <http://www.w3.org/2001/XMLSchema#>
PREFIX :<http://www.semanticweb.org/zhenyuzhang/ontologies/2021/DREAMDNPTO#>
SELECT ?internationalised_resource_identifier_short_name_of_manifestation ?label_of_manifestation
            WHERE { ?internationalised_resource_identifier_short_name_of_manifestation rdfs:subClassOf*
:ManifestationOfEmotionalAndMoodDisturbanceInDementia;
rdfs:label ?label_of_manifestation. } ORDER BY ?label_of_manifestation
```

internationalised_resource_identifier_short_name_of_manifestation	label_of_manifestation
AutonomicHyperactivity	"Autonomic Hyperactivity"@en
Avoidance	"Avoidance"@en
BehavioralInaction	"Behavioral Inaction"@en
BeingOverlyFamiliarWithOtherPeople	"Being Overly Familiar with Other People"@en
BeingUnusuallyEmotional	"Being Unusually Emotional"@en
CompulsiveSexualBehaviour	"Compulsive Sexual Behaviour"@en
DecreasedNeedForSleep	"Decreased Need for Sleep"@en
DemandingBehaviour	"Demanding Behaviour"@en
DiminishedAbilityOfConcentration	"Diminished Ability of Concentration"@en
DiminishedAbilityOfThinking	"Diminished Ability of Thinking"@en
DowncastGaze	"Downcast Gaze"@en
Dysphoria	"Dysphoria"@en
ExcessiveWorry	"Excessive Worry"@en
Fear	"Fear"@en
FearOfBathing	"Fear of Bathing"@en
FearOfBeingAbandoned	"Fear of Being Abandoned"@en
FearOfBeingAlone	"Fear of Being Alone"@en
FearOfCrowd	"Fear of Crowd"@en
FearOfDark	"Fear of Dark"@en
FearOfFalling	"Fear of Falling"@en
FearOfTransferring	"Fear of Transferring"@en
FeelingDiscouraged	"Feeling Discouraged"@en
FeelingEmpty	"Feeling Empty"@en
FeelingHopeless	"Feeling Hopeless"@en
FeelingSad	"Feeling Sad"@en
FeelingWorthless	"Feeling Worthless"@en
FeelingOfExcessiveGuilt	"Feeling of Excessive Guilt"@en
GodotSyndrome	"Godot Syndrome"@en
Indecisiveness	"Indecisiveness"@en
IndifferentEmotion	"Indifferenct Emotion"@en
Insensitivity	"Insensitivity"@en
JokingExcessively	"Joking Excessively"@en
LackOfConcern	"Lack of Concern"@en
LackOfFeelingEmotion	"Lack of Feeling Emotion"@en
LackOfSpontaneity	"Lack of Spontaneity"@en
LaughingExcessively	"Laughing Excessively"@en
LessAttentionToSurroundingEnvironment	"Less Attention to Surrounding Environment"@en
LessLikelyToEngageInConversation	"Less Likely to Engage in Conversation"@en
LessLikelyToInitiateConversation	"Less Likely to Initiate Conversation"@en
LossOfEnergy	"Loss of Energy"@en
LossOfInterest	"Loss of Interest"@en
LossOfInterestInEatingFood	"Loss of Interest in Eating Food"@en
LossOfInterestInFamily	"Loss of Interest in Family"@en
LossOfInterestInFriend	"Loss of Interest in Friend"@en
LossOfInterestInPreviouslyEnjoyableActivity	"Loss of Interest in Previously Enjoyable Activity"@en
LossOfLibido	"Loss of Libido"@en
LossOfMotivation	"Loss of Motivation"@en
LossOfPleasure	"Loss of Pleasure"@en
LossOfPleasureFromUsuallyPleasuableActivity	"Loss of Pleasure from Usually Pleasuable Activity"@en

LossOfSelfEsteem	"Loss of Self-Esteem"@en
ManifestationOfAnxietyInDementia	"Manifestation of Anxiety in Dementia"@en
ManifestationOfApathyInDementia	"Manifestation of Apathy in Dementia"@en
ManifestationOfDepressionInDementia	"Manifestation of Depression in Dementia"@en
ManifestationOfEmotionalAndMoodDisturbanceInDementia	"Manifestation of Emotional and Mood Disturbance in Dementia"@e
ManifestationOfEuphoriaInDementia	"Manifestation of Euphoria in Dementia"@en
MotorTension	"Motor Tension"@en
Panic	"Panic"@en
PessimisticThought	"Pessimistic Thought"@en
PressureOfSpeech	"Pressure of Speech"@en
PsychosomaticSymptom	"Psychosomatic Symptom"@en
RacingThoughts	"Racing Thoughts"@en
ReducedAffection	"Reduced Affection"@en
ReducedInitiation	"Reduced Initiation"@en
ReducedWillingnessToMakeEffort	"Reduced Willingness to Make Effort"@en
RefusingFood	"Refusing Food"@en
RiskTakingBehaviour	"Risk-Taking Behaviour"@en
SelfIsolation	"Self Isolation"@en
SelfNeglect	"Self Neglect"@en
SuicidalIdeation	"Suicidal Ideation"@en
Suicide	"Suicide"@en
SuicideAttempt	"Suicide Attempt"@en
Tearfulness	"Tearfulness"@en
Withdrawal	"Withdrawal"@en

SPARQL query and query results for competency question No.4 “What are the main activities for managing emotional and mood disturbances of people with dementia in living long-term care facilities?”

SPARQL query:

```
PREFIX rdf: <http://www.w3.org/1999/02/22-rdf-syntax-ns#>
PREFIX owl: <http://www.w3.org/2002/07/owl#>
PREFIX rdfs: <http://www.w3.org/2000/01/rdf-schema#>
PREFIX xsd: <http://www.w3.org/2001/XMLSchema#>
PREFIX :<http://www.semanticweb.org/zhenyuzhang/ontologies/2021/DREAMDNPTO#>
SELECT ?internationalised_resource_identifier_short_name_of_management_activity ?label_of_management_activity
        WHERE { ?internationalised_resource_identifier_short_name_of_management_activity rdfs:subClassOf*
:ManagementActivityOfEmotionalAndMoodDisturbanceInDementia;
rdfs:label ?label_of_management_activity. } ORDER BY ?label_of_management_activity
```

internationalised_resource_identifier_short_name_of_management_activity	label_of_management_activity
ActiveMusicTherapy	"Active Music Therapy"@en
Acupressure	"Acupressure"@en
AdministrationOfMedication	"Administration of Medication"@en
AerobicExercise	"Aerobic Exercise"@en
AnimalAssistedTherapy	"Animal Assisted Therapy"@en
ApplyingCommunicationSkill	"Applying Communication Skill"@en
ApplyingDressingToWound	"Applying Dressing to Wound"@en
ApplyingHotPack	"Applying Hot Pack"@en
ApplyingWarmBlanket	"Applying Warm Blanket"@en
AquaticExercise	"Aquatic Exercise"@en
ArmchairTravelActivity	"Armchair Travel Activity"@en
Aromatherapy	"Aromatherapy"@en
ArtTherapy	"Art Therapy"@en
AssistingInApplyingMakeup	"Assisting in Applying Makeup"@en
AssistingInChoosingAppropriateClothing	"Assisting in Choosing Appropriate Clothing"@en
AssistingInCuttingUpFood	"Assisting in Cutting up Food"@en
AssistingInFeedingPerson	"Assisting in Feeding Person"@en
AssistingInPuttingOnClothes	"Assisting in Putting on Clothes"@en
AssistingInTakingOffClothes	"Assisting in Taking off Clothes"@en
AssistingWithActivityOfDailyLiving	"Assisting with Activity of Daily Living"@en
AssistingWithContinenceCare	"Assisting with Continence Care"@en
AssistingWithEating	"Assisting with Eating"@en
AssistingWithHairCare	"Assisting with Hair Care"@en
AssistingWithMedicationManagement	"Assisting with Medication Management"@en
AssistingWithMobility	"Assisting with Mobility"@en
AssistingWithNailCare	"Assisting with Nail Care"@en
AssistingWithOralCare	"Assisting with Oral Care"@en
AssistingWithPersonalGrooming	"Assisting with Personal Grooming"@en
AssistingWithPersonalHygiene	"Assisting with Personal Hygiene"@en
AssistingWithSleep	"Assisting with Sleep"@en
AssistingWithToileting	"Assisting with Toileting"@en
AttendingConcert	"Attending Concert"@en
AttendingVirtualEvent	"Attending Virtual Event"@en
AttendingVirtualMuseumTour	"Attending Virtual Museum Tour"@en
AttendingVirtualZooTour	"Attending Virtual Zoo Tour"@en
BPSDAssessmentTraining	"BPSD Assessment Training"@en
BalanceExercise	"Balance Exercise"@en
BalloonGame	"Balloon Game"@en
BarbecueActivity	"Barbecue Activity"@en
BehaviourTherapy	"Behaviour Therapy"@en
BehaviouralAssessment	"Behavioural Assessment"@en
BehaviouralObservation	"Behavioural Observation"@en
BibleReading	"Bible Reading"@en
BiographicallyOrientatedMobilisation	"Biographically Orientated Mobilisation"@en
BreathingExercise	"Breathing Exercise"@en
BrightWhiteLightTherapy	"Bright White Light Therapy"@en
BuddySystemIntervention	"Buddy System Intervention"@en
BusTrip	"Bus Trip"@en
CardGame	"Card Game"@en

CasinoActivity	"Casino Activity"@en
CausativeFactorInvestigation	"Causative Factor Investigation"@en
CelebrationActivity	"Celebration Activity"@en
ChangingContinenceAid	"Changing Continence Aid"@en
CheckingHearingAid	"Checking Hearing Aid"@en
CheckingPerson	"Checking Person"@en
CheckingVisionAid	"Checking Vision Aid"@en
ChurchServiceActivity	"Church Service Activity"@en
CleaningHearingAid	"Cleaning Hearing Aid"@en
CleaningVisionAid	"Cleaning Vision Aid"@en
CoffeeClubActivity	"Coffee Club Activity"@en
CognitiveAssessment	"Cognitive Assessment"@en
CognitiveBehaviouralTherapy	"Cognitive Behavioural Therapy"@en
CognitiveRehabilitation	"Cognitive Rehabilitation"@en
CognitiveStimulationTherapy	"Cognitive Stimulation Therapy"@en
CognitiveTherapy	"Cognitive Therapy"@en
CognitiveTraining	"Cognitive Training"@en
ColouringPicture	"Colouring Picture"@en
CommunicationSkillTraining	"Communication Skill Training"@en
ComputerisedCognitiveBehaviourTherapy	"Computerised Cognitive Behavioural Therapy"@en
ConsistencyOfStaff	"Consistency of Staff Providing Care"@en
Cooking	"Cooking"@en
Counselling	"Counselling"@en
CreativePursuitActivity	"Creative Pursuit Activity"@en
CrushingOralMedicationForPeopleWithSwallowingDifficulty	"Crushing Oral Medication for People with Swallowing Difficulty"@en
DanceTherapy	"Dance Therapy"@en
DeepBreathingExercise	"Deep Breathing Exercise"@en
DeliriumAssessment	"Delirium Assessment"@en
DescribingBaselineBehaviour	"Describing Baseline Behaviour"@en
DescribingChangedBehaviour	"Describing Changed Behaviour"@en
DifferentialDiagnosis	"Differential Diagnosis"@en
Distraction	"Distraction"@en
DiversionalTherapy	"Diverstional Therapy"@en
DollTherapy	"Doll Therapy"@en
EmotionalSupport	"Emotional Support"@en
EmotionalAndMoodDisturbanceEducationToFamilyMember	"Emotional and Mood Disturbance Education to Family Member"@e
EncouragingCommunication	"Encouraging Communication"@en
EncouragingPhysicalActivity	"Encouraging Physical Activity"@en
EncouragingToDrinkWater	"Encouraging to Drink Water"@en
EncouragingToEatFood	"Encouraging to Eat Food"@en
EngagingPersonInActivity	"Engaging Person in Activity"@en
EnhancingVisualAccess	"Enhancing Visual Access"@en
EnvironmentalModification	"Environmental Modification"@en
Ergotherapy	"Ergotherapy"@en
Evaluation	"Evaluation"@en
ExplanationOfNursingCare	"Explanation of Nursing Care"@en
FamilyMemberFocusedIntervention	"Family Member Focused Intervention"@en
FamilyMemberVisit	"Family Member Visit"@en
FlexibilityExercise	"Flexibility Exercise"@en
FloorGame	"Floor Game"@en
FoldingClothes	"Folding Clothes"@en
FoldingTowel	"Folding Towel"@en
FootyTippingActivity	"Footy Tipping Activity"@en
FriendVisit	"Friend Visit"@en
Gardening	"Gardening"@en
GetIntoReading	"Get into Reading"@en
GivingEncouragement	"Giving Encouragement"@en
GolfPuttingGame	"Golf Putting Game"@en
HairAppointment	"Hair Appointment"@en
Handicraft	"Handicraft"@en
HappyHourActivity	"Happy Hour Activity"@en
HavingGarden	"Having Garden"@en
HavingGardenShed	"Having Garden Shed"@en

HavingKitchen	"Having Kitchen"@en
HavingPersonalFurnishing	"Having Personal Furnishing"@en
HavingPersonalMemoryBox	"Having Personal Memory Box"@en
HavingPersonalisedDoor	"Having Personalised Door"@en
HavingRegularRoutine	"Having Regular Routine"@en
HavingWashingLine	"Having Washing Line"@en
HealthAssessment	"Health Assessment"@en
HealthCareProfessionalFocusedIntervention	"Health Care Professional Focused Intervention"@en
HealthCareProfessionalRecognitionProgram	"Health Care Professional Recognition Program"@en
Homeopathy	"Homeopathy"@en
HorticulturalTherapy	"Horticultural Therapy"@en
HumorTherapy	"Humor Therapy"@en
IdentifyingAntecedentOfBehaviour	"Identifying Antecedant of Behaviour"@en
IdentifyingCareGoal	"Identifying Care Goal"@en
IdentifyingConsequenceOfBehaviour	"Identifying Consequence of Behaviour"@en
IdentityingLocationOfBehaviour	"Identifying Location of Behaviour"@en
IdentifyingPeopleAffected	"Identifying People Affected"@en
IdentifyingPeopleInvolved	"Identifying People Involved"@en
IdentifyingTimeOfBehaviour	"Identifying Time of Behaviour"@en
IdentifyingWitness	"Identifying Witness"@en
InformingLegalRepresentative	"Informing Legal Representative"@en
InformingMedicalPractitioner	"Informing Medical Practitioner"@en
InformingNurse	"Informing Nurse"@en
InformingRelative	"Informing Relative"@en
InteractionWithOtherCareRecipient	"Interaction with Other Care Recipient"@en
IntergenerationalCare	"Intergenerational Care"@en
InternetBasedIntervention	"Internet-Based Intervention"@en
InterpersonalPsychotherapy	"Interpersonal Psychotherapy"@en
Intervention	"Intervention"@en
InvolvingCareRecipientInCarePlanning	"Involving Care Recipient in Care Planning"@en
InvolvingLegalRepresentativeInCarePlanning	"Involving Legal Representative in Care Planning"@en
JapaneseGardenIntervention	"Japanese Garden Intervention"@en
KnittingAcitivity	"Knitting Activity"@en
LegoActivity	"Lego Activity"@en
LiaisingWithLegalRepresentative	"Liaising with Legal Representative"@en
LiaisingWithMedicalPractitioner	"Liaising with Medical Practitioner"@en
LiaisingWithPharmacist	"Liaising with Pharmacist"@en
LiasingWithRelative	"Liaising with Relative"@en
LightTherapy	"Light Therapy"@en
ListeningToAudiobook	"Listening to Audiobook"@en
ListeningToClassicalMusic	"Listening to Classical Music"@en
ListeningToFamiliarMusic	"Listening to Familiar Music"@en
ListeningToPreferredMusic	"Listening to Preferred Music"@en
ListeningToRadio	"Listening to Radio"@en
ListeningToRelaxingMusic	"Listening to Relaxing Music"@en
LockingUpMedicationWhenNotInUse	"Locking Up Medication When not in Use"@en
MakingBed	"Making Bed"@en
MakingBuzzerEasyToReach	"Making Buzzer Easy to Reach"@en
MakingExitLessObvious	"Making Exit Less Obvious"@en
ManagementActivityOfEmotionalAndMoodDisturbanceInDementia	"Management Activity of Emotional and Mood Disturbance in Deme
MassageTherapy	"Massage Therapy"@en
MatchingSocks	"Matching Socks"@en
MemoryTraining	"Memory Training"@en
MindBodyTherapy	"Mind-Body Therapy"@en
MiniMartActivity	"Mini Mart Activity"@en
Monitoring	"Monitoring"@en
MonitoringPersonTakingMedication	"Monitoring Person Taking Medication"@en
MontessoriIntervention	"Montessori Intervention"@en
MultisensoryStimulation	"Multisensory Stimulation"@en
MusicTherapeuticCaregiving	"Music Therapeutic Caregiving"@en
MusicTherapy	"Music Therapy"@en
NatureOfBPSDTraining	"Nature of BPSD Training"@en
NeurologicalAssessment	"Neurological Assessment"@en

NoiseControl	"Noise Control"@en
NonpharmacologicalIntervention	"Nonpharmacological Intervention"@en
NonpharmacologicalInterventionTraining	"Nonpharmacological Intervention Training"@en
NursingPracticeIntervention	"Nursing Practice Intervention"@en
Observation	"Observation"@en
ObtainingInformedConsent	"Obtaining Informed Consent"@en
OccupationalTherapy	"Occupational Therapy"@en
OfferingDrink	"Offering Drink"@en
OfferingFood	"Offering Food"@en
OneOnOnePersonalContact	"One on One Interaction"@en
PRIDEBathingApproach	"PRIDE Bathing Approach"@en
PainAssessment	"Pain Assessment"@en
PaintingPicture	"Painting Picture"@en
PamperGroupActivity	"Pamper Group Activity"@en
ParticipatingActiveMusicTherapyByMovingBody	"Participating Active Music Therapy by Moving Body"@en
ParticipatingActiveMusicTherapyByPlayingMusicalInstrument	"Participating Active Music Therapy by Playing Musical Instrument"@
ParticipatingActiveMusicTherapyBySinging	"Participating Active Music Therapy by Singing"@en
ParticipatingActiveMusicTherapyByWritingSong	"Participating Active Music Therapy by Writing Song"@en
PassiveMusicTherapy	"Passive Music Therapy"@en
PastoralCare	"Pastoral Care"@en
PayingCloseAttentionToSharpObject	"Paying Close Attention to Sharp Object"@en
PersonCentredCareEudcationToHealthCareProfessional	"Person Centred Care Education to Health Care Professional"@en
PersonWithDementiaFocusedIntervention	"Person with Dementia Focused Intervention"@en
PetTherapy	"Pet Therapy"@en
PharmacologicalIntervention	"Pharmacological Intervention"@en
PhoneCall	"Phone Call"@en
PhysicalEnvironmentalIntervention	"Physical Environmental Intervention"@en
PhysicalExamination	"Physical Examination"@en
PhysicalExerciseTherapy	"Physical Exercise Therapy"@en
PhysicalRestraint	"Physical Restraint"@en
Physiotherapy	"Physiotherapy"@en
PlayingBallGame	"Playing Ball Game"@en
PlayingBingoGame	"Playing Bingo Game"@en
PlayingBoardGame	"Playing Board Game"@en
PlayingBowling	"Playing Bowling"@en
PlayingCarpetBowels	"Playing Carpet Bowels"@en
PlayingChairVolleyball	"Playing Chair Volleyball"@en
PlayingVideoGame	"Playing Video Game"@en
PoetryTherapy	"Poetry Therapy"@en
PolishingFavouriteObject	"Polishing Favourite Object"@en
PoppingBubbleWrap	"Popping Bubble Wrap"@en
ProblemSolvingTherapy	"Problem Solving Therapy"@en
ProvidingActivityArea	"Providing Activity Area"@en
ProvidingAdequateRest	"Providing Adequate Rest"@en
ProvidingCaffeineFreeBeverage	"Providing Caffeine-Free Beverage"@en
ProvidingCalmEnvironment	"Providing Calm Environment"@en
ProvidingClearMarkedAccessToBathroom	"Providing Clear Marked Access to Bathroom"@en
ProvidingClearMarkedAccessToPersonalBelonging	"Providing Clear Marked Access to Personal Belonging"@en
ProvidingConcentratedOxygen	"Providing Concentrated Oxygen"@en
ProvidingContinenceAid	"Providing Continence Aid"@en
ProvidingCrashMatForBedsideSafety	"Providing Crash Mat for Bedside Safety"@en
ProvidingDryingRackInRoom	"Providing Drying Rack In Room"@en
ProvidingEasyAccessToOutsideSpace	"Providing Easy Access to Outside Space"@en
ProvidingFavouriteFood	"Providing Favourite Food"@en
ProvidingHoist	"Providing Hoist"@en
ProvidingHomeLikeEnvironment	"Providing Home Like Environment"@en
ProvidingPrivateBathroom	"Providing Private Bathroom"@en
ProvidingSafeOutsideSpace	"Providing Safe Outside Space"@en
ProvidingSensorMat	"Providing Sensor Mat"@en
ProvidingSingleBedroom	"Providing Single Bedroom"@en
ProvidingSnack	"Providing Snack"@en
ProvidingSufficientLighting	"Providing Sufficient Lighting"@en
ProvidingAVarietyOfSpaceInEnvironment	"Providing a Variety of Space in Environment"@en

Psychoeducation	"Psychoeducation"@en
Psychotherapy	"Psychotherapy"@en
ReadingActivity	"Reading Activity"@en
ReadingBook	"Reading Book"@en
ReadingMagazine	"Reading Magazine"@en
ReadingNewspaper	"Reading Newspaper"@en
RealityOrientationTherapy	"Reality Orientation Therapy"@en
Reassurance	"Reassurance"@en
RecreationalTherapy	"Recreational Therapy"@en
Redirection	"Redirection"@en
ReducingClutter	"Reducing Clutter"@en
ReducingLightGlare	"Reducing Light Glare"@en
ReducingNoiseOfNonemergencyOverheadPaging	"Reducing Noise of Non-emergency Overhead Paging"@en
ReductionOfDaytimeSleep	"Reduction of Daytime Sleep"@en
ReferralToPolice	"Referral to Police"@en
ReferralToPsychiatrist	"Referral to Psychiatrist"@en
ReferralToPsychogeriatration	"Referral to Psychogeriatration"@en
ReferralToPsychologist	"Referral to Psychologist"@en
ReferralToRequiredServiceProvider	"Referral to Required Service Provider"@en
ReferralToASpecialist	"Referral to a Specialist"@en
Reflexology	"Reflexology"@en
RegularToileting	"Regular Toileting"@en
RelaxationTherapy	"Relaxation Therapy"@en
RelocatingPersonToAppropriateArea	"Relocating Person to Appropirate Area"@en
Reminding	"Reminding"@en
ReminiscenceTherapy	"Reminiscence Therapy"@en
RemovingDangerousObject	"Removing Dangerous Object"@en
Reorientation	"Reorientation"@en
ReorientationToEvent	"Reorientation to Event"@en
ReorientationToPerson	"Reorientation to Person"@en
ReorientationToPlace	"Reorientation to Place"@en
ReorientationToTime	"Reorientation to Time"@en
Repositioning	"Repositioning"@en
ReviewOfComplementaryMedication	"Review of Complementary Medication"@en
ReviewOfFamilyHistoryOfNeurologicalDisorder	"Review of Family History of Neurological Disorder"@en
ReviewOfFamilyHistoryOfPsychiatricDisorder	"Review of Family History of Psychiatric Disorder"@en
ReviewOfMedicalRecord	"Review of Medical Record"@en
ReviewOfMedication	"Review of Medication"@en
ReviewOfNursingRecord	"Review of Nursing Record"@en
ReviewOfNutraceutical	"Review of Nutraceutical"@en
ReviewOfPastMedicalHistory	"Review of Past Medical History"@en
ReviewOfPathologyTest	"Review of Pathology Test"@en
ReviewOfPrescribedMedication	"Review of Prescribed Medication"@en
ReviewOfPriorGeriatricEvaluationResult	"Review of Prior Geriatric Evaluation Result"@en
ReviewOfPriorNeuroimagingStudyResult	"Review of Prior Neuroimaging Study Result"@en
ReviewOfPriorNeurologicalEvaluationResult	"Review of Prior Neurological Assessment Result"@en
ReviewOfPriorNeuropsychologicalTestResult	"Review of Prior Neuropsychological Test Result"@en
ReviewOfPriorPsychiatricEvaluationResult	"Review of Prior Psychiatric Evaluation Result"@en
ReviewOfOverTheCounterMedication	"Review of over the Counter Medication"@en
RiskAssessment	"Risk Assessment"@en
RoboticAnimalAssistedTherapy	"Robotic Animal-Assisted Therapy"@en
ScheduledReassuranceTherapy	"Scheduled Reassurance Therapy"@en
SchedulingChallengingActivityWhenPersonIsAtHisBest	"Scheduling Challenging Activity When Person with Dementia is at H
ScreeningForDrivingSafety	"Screening for Driving Safety"@en
ScreeningForFoodPreparationSafety	"Screening for Food Preparation Safety"@en
SelfMaintenanceTherapy	"Self-Maintenance Therapy"@en
SensoryStimulationTherapy	"Sensory Stimulation Therapy"@en
SewingActivity	"Sewing Activity"@en
SimplifyingTask	"Simplifying Task"@en
SimulatedPresenceTherapy	"Simulated Presence Therapy"@en
SittingAndTalkingWithPerson	"Sitting and Talking with Person"@en
SmellingFlower	"Smelling Flower"@en
SnoezolenMultisensoryStimulationTherapy	"Snoezelen Multisensory Stimulation Therapy"@en

SocialEnvironmentalIntervention	"Social Environmental Intervention"@en
SocialInteractionIntervention	"Social Interaction Intervention"@en
SocialMediaVideoCall	"Social Media Video Call"@en
SocialOuting	"Social Outing"@en
SocialSupportGroupProgram	"Social Support Group Program"@en
SolvingWordSearchPuzzle	"Solving Word Search Puzzle"@en
SonasIntervention	"Sonas Intervention"@en
SortingButton	"Sorting Button"@en
SortingCoin	"Sorting Coin"@en
SpiritualSupport	"Spiritual Support"@en
SpiritualTherapy	"Spiritual Therapy"@en
StaffSupervision	"Staff Supervision"@en
Storytelling	"Storytelling"@en
StrengthExercise	"Strength Exercise"@en
StretchingExercise	"Stretching Exercise"@en
StructuredActivity	"Structured Activity"@en
SunlightTherapy	"Sunlight Therapy"@en
SupportToFamilyMember	"Support to Family Member"@en
SupportToHealthCareProfessional	"Support to Health Care Professional"@en
Swimming	"Swimming"@en
TaiChi	"Tai Chi"@en
TakingVitalSign	"Taking Vital Sign"@en
TastingSweetFood	"Tasting Sweet Food"@en
TenderLovingCare	"Tender Loving Care"@en
FloorGame	"The game that can be played on floors, e.g., floor chess game."@[illegible]
TherapyForEmotionalAndMoodDisturbanceInDementia	"Therapy for Emotional and Mood Disturbance in Dementia"@en
TouchTherapy	"Touch Therapy"@en
ToyAnimalAssistedTherapy	"Toy Animal-Assisted Therapy"@en
TransferOfCare	"Transfer of Care"@en
TransferOfCareToAnotherLongTermCareFacility	"Transfer of Care to Another Long Term Care Facility"@en
TransferOfCareToHome	"Transfer of Care to Home"@en
TransferOfCareToHospital	"Transfer of Care to Hospital"@en
TreatmentPlanDevelopment	"Treatment Plan Development"@en
TurningOffTelevision	"Turning off Television"@en
Urinalysis	"Urinalysis"@en
UsingCueCard	"Using Cue Card"@en
UsingSocialRobot	"Using Social Robot"@en
UsingUnobtrusiveSafetyDesignForPhysicalEnviornment	"Using Unobtrusive Safety Design for Physical Environment"@en
UsingVerbalReminder	"Using Verbal Reminder"@en
UsingWorryBeads	"Using Worry Beads"@en
UsingWrittenNoteAsReminder	"Using Written Note as Reminder"@en
UsingPortableBedsideCommode	"Using a Portable Bedside Commode"@en
ValidationTherapy	"Validation Therapy"@en
VirtualRealityTherapy	"Virtual Reality Therapy"@en
VolunteerProgram	"Volunteer Program"@en
Walking	"Walking"@en
WatchingMovie	"Watching Moive"@en
WatchingTelevision	"Watching Television"@en
WoodworkingActivity	"Woodworking Activity"@en
WorkforcePlanning	"Workforce Planning"@en
Yoga	"Yoga"@en

SPARQL query and query results for competency question No.5 “What tools are used to measure emotional and mood disturbances in people with dementia living in long-term care facilities?”

SPARQL query:

```
PREFIX rdf: <http://www.w3.org/1999/02/22-rdf-syntax-ns#>
PREFIX owl: <http://www.w3.org/2002/07/owl#>
PREFIX rdfs: <http://www.w3.org/2000/01/rdf-schema#>
PREFIX xsd: <http://www.w3.org/2001/XMLSchema#>
PREFIX :<http://www.semanticweb.org/zhenyuzhang/ontologies/2021/DREAMDNPTO#>
SELECT ?internationalised_resource_identifier_short_name_of_assessment_tool ?label_of_assessment_tool
            WHERE { ?internationalised_resource_identifier_short_name_of_assessment_tool rdfs:subClassOf*
:AssessmentToolForEmotionalAndMoodDisturbanceInDementia;
rdfs:label ?label_of_assessment_tool. } ORDER BY ?label_of_assessment_tool
```

internationalised_resource_identifier_short_name_of_assessment_tool	label_of_assessment_tool
AlzheimersDiseaseRelatedDisordersMoodScale	"Alzheimer's Disease and Related Dementia Mood Scale"@en
ApathyEvaluationScale	"Apathy Evaluation Scale"@en
ApathyEvaluationScaleClinicianVersion	"Apathy Evaluation Scale Clinician Version"@en
ApathyEvaluationScaleInformantVersion	"Apathy Evaluation Scale Informant Version"@en
ApathyEvaluationScaleSelfVersion	"Apathy Evaluation Scale Self Version"@en
ApathyInventory	"Apathy Inventory"@en
ApathyInDementiaNursingHomeScale	"Apathy in Dementia Nursing Home Scale"@en
AssessmentToolForEmotionalAndMoodDisturbanceInDementia	"Assessment Tool for Emotional and Mood Disturbance in Dementia'
BeckDepressionInventory	"Beck Depression Inventory"@en
BehavioralPathologyInAlzheimersDiseaseRatingScale	"Behavioral Pathology in Alzheimer's Disease Rating Scale"@en
BehaviourRatingScaleForDementia	"Behaviour Rating Scale for Dementia"@en
BondLaderVisualAnalogueScale	"Bond-Lader Visual Analogue Scale"@en
BriefPsychiatricRatingScale	"Brief Psychiatric Rating Scale"@en
CenterForEpidemiologicalStudiesDepressionScale	"Center for Epidemiological Studies Depression Scale"@en
CornellScaleForDepressionInDementia	"Cornell Scale for Depression in Dementia"@en
DementiaApathyInterviewAndRating	"Dementia Apathy Interview and Rating"@en
DementiaMoodAssessmentScale	"Dementia Mood Assessment Scale"@en
DementiaMoodPictureTest	"Dementia Mood Picture Test"@en
DimensionalApathyScale	"Dimensional Apathy Scale"@en
GeriatricAnxietyInventory	"Geriatric Anxiety Inventory"@en
GeriatricDepressionScale	"Geriatric Depression Scale"@en
GeriatricDepressionScaleShortForm	"Geriatric Depression Scale Short Form"@en
GorrfriesBraneSteenScale	"Gottfries-Bråne-Steen Scale"@en
HamiltonAnxietyRatingScale	"Hamilton Anxiety Rating Scale"@en
HamiltonRatingScaleForDepression	"Hamilton Rating Scale for Depression"@en
HospitalAnxietyAndDepressionScale	"Hospital Anxiety and Depression Scale"@en
KurzSkalaStimmungAktivierung	"KurzSkala Stimmung/Aktivierung"@en
LilleApathyRatingScale	"Lille Apathy Rating Scale"@en
MiniInternationalNeuropsychiatricInterview	"Mini International Neuropsychiatric Interview"@en
MiniMentalStateExamination	"Mini-Mental Sate Examination"@en
MontgomeryAsbergDepressionRatingScale	"Montgomery-Asberg Depression Rating Scale"@en
MultidimensionalObservationScaleForElderlySubjects	"Multidimensional Observation Scale for Elderly Subjects"@en
NeuropsychiatricInventory	"Neuropsychiatric Inventory"@en
NeuropsychiatricInventoryClinicianRatingScale	"Neuropsychiatric Inventory - Clinician Rating Scale"@en
NeuropsychiatricInventoryNursingHomeVersion	"Neuropsychiatric Inventory - Nursing Home Version"@en
ObservedEmotionRatingScale	"Observed Emotion Rating Scale"@en
PassivityInDementiaScale	"Passivity in Dementia Scale"@en
PatientHealthQuestionnaire9	"Patient Health Questionnaire - 9"@en
PhiladelphiaGeriatricCenterAffectRatingScale	"Philadelphia Geriatric Center Affect Rating Scale"@en
RatingAnxietyInDementia	"Rating Anxiety in Dementia"@en
RevisedMemoryAndBehaviorProblemsChecklist	"Revised Memory and Behavior Problems Checklist"@en
SaintLouisUniversityMentalStatusExamination	"Saint Louis University Mental Status Examination"@en
SandozClinicalAssessmentGeriatricScale	"Sandoz Clinical Assessment - Geriatric Scale"@en
ScaleForTheAssessmentOfNegativeSymptomsInAlzheimersDisease	"Scale for the Assessment of Negative Symptoms in Alzheimer's Dise
StarksteinApathyScale	"Starkstein Apathy Scale"@en
StateTraitAnxietyInventory	"State-Trait Anxiety Inventory"@en
StateTraitAnxietyInventoryForAdults	"State-Trait Anxiety Inventory for Adults"@en
VisualAnalogMoodScale	"Visual Analog Mood Scale"@en
WorryScale	"Worry Scale"@en

SPARQL query and query results for competency question No.6 “What are the care goals for managing emotional and mood disturbances of people with dementia living in long-term care facilities?”

SPARQL query:

```
PREFIX rdf: <http://www.w3.org/1999/02/22-rdf-syntax-ns#>
PREFIX owl: <http://www.w3.org/2002/07/owl#>
PREFIX rdfs: <http://www.w3.org/2000/01/rdf-schema#>
PREFIX xsd: <http://www.w3.org/2001/XMLSchema#>
PREFIX :<http://www.semanticweb.org/zhenyuzhang/ontologies/2021/DREAMDNPTO#>
SELECT ?internationalised_resource_identifier_short_name_of_care_goal ?label_of_care_goal
            WHERE { ?internationalised_resource_identifier_short_name_of_care_goal rdfs:subClassOf*
:CareGoalForEmotionalAndMoodDisturbanceInDementia;
rdfs:label ?label_of_care_goal. } ORDER BY ?label_of_care_goal
```

internationalised_resource_identifier_short_name_of_care_goal	label_of_care_goal
AbsenceOfEmotionalAndMoodDisturbanceRelevantSymptom	"Absence of Emotional and Mood Disturbance Relevant Symptom"@en
CareGoalForEmotionalAndMoodDisturbanceInDementia	"Care Goal for Emotional and Mood Disturbance in Dementia"@en
ImprovingQualityOfLifeOfOtherPeople	"Improving Quality of Life of Other People"@en
ImprovingQualityOfLifeOfPersonWithEmotionalAndMoodDisturbance	"Improving Quality of Life of Person with Emotional and Mood Disturbance"@en
ReducingFrequencyOfEmotionalAndMoodDisturbance	"Reducing Frequency of Emotional and Mood Disturbance"@en
ReducingHarmToOtherPeople	"Reducing Harm to Other People"@en
ReducingHarmToSelf	"Reducing Harm to Self"@en
ReducingSeverityOfEmotionalAndMoodDisturbance	"Reducing Severity of Emotional and Mood Disturbance"@en
StabilisingEmotionalAndMoodDisturbance	"Stabilising Emotional and Mood Disturbance"@en

SPARQL query and query results for competency question No.7 “What non-pharmacological interventions are used for emotional and mood disturbances of people with dementia living in long-term care facilities?”

SPARQL query:

```
PREFIX rdf: <http://www.w3.org/1999/02/22-rdf-syntax-ns#>
PREFIX owl: <http://www.w3.org/2002/07/owl#>
PREFIX rdfs: <http://www.w3.org/2000/01/rdf-schema#>
PREFIX xsd: <http://www.w3.org/2001/XMLSchema#>
PREFIX :<http://www.semanticweb.org/zhenyuzhang/ontologies/2021/DREAMDNPTO#>
SELECT ?internationalised_resource_identifier_short_name_of_nonpharmacological_intervention
?label_of_nonpharmacological_intervention
            WHERE { ?internationalised_resource_identifier_short_name_of_nonpharmacological_intervention rdfs:subClassOf*
:NonpharmacologicalIntervention;
rdfs:label ?label_of_nonpharmacological_intervention. } ORDER BY ?label_of_nonpharmacological_intervention
```

internationalised_resource_identifier_short_name_of_nonpharmacological_intervention	label_of_nonpharmacological_intervention
ActiveMusicTherapy	"Active Music Therapy"@en
Acupressure	"Acupressure"@en
AdministrationOfMedication	"Administration of Medication"@en
AerobicExercise	"Aerobic Exercise"@en
AnimalAssistedTherapy	"Animal Assisted Therapy"@en
ApplyingDressingToWound	"Applying Dressing to Wound"@en
ApplyingHotPack	"Applying Hot Pack"@en
ApplyingWarmBlanket	"Applying Warm Blanket"@en
AquaticExercise	"Aquatic Exercise"@en
ArmchairTravelActivity	"Armchair Travel Activity"@en
Aromatherapy	"Aromatherapy"@en
ArtTherapy	"Art Therapy"@en
AssistingInApplyingMakeup	"Assisting in Applying Makeup"@en
AssistingInChoosingAppropriateClothing	"Assisting in Choosing Appropriate Clothing"@en
AssistingInCuttingUpFood	"Assisting in Cutting up Food"@en
AssistingInFeedingPerson	"Assisting in Feeding Person"@en
AssistingInPuttingOnClothes	"Assisting in Putting on Clothes"@en
AssistingInTakingOffClothes	"Assisting in Taking off Clothes"@en
AssistingWithActivityOfDailyLiving	"Assisting with Activity of Daily Living"@en
AssistingWithContinenceCare	"Assisting with Continence Care"@en
AssistingWithEating	"Assisting with Eating"@en
AssistingWithHairCare	"Assisting with Hair Care"@en
AssistingWithMedicationManagement	"Assisting with Medication Management"@en
AssistingWithMobility	"Assisting with Mobility"@en
AssistingWithNailCare	"Assisting with Nail Care"@en
AssistingWithOralCare	"Assisting with Oral Care"@en
AssistingWithPersonalGrooming	"Assisting with Personal Grooming"@en
AssistingWithPersonalHygiene	"Assisting with Personal Hygiene"@en
AssistingWithSleep	"Assisting with Sleep"@en
AssistingWithToileting	"Assisting with Toileting"@en
AttendingConcert	"Attending Concert"@en
AttendingVirtualEvent	"Attending Virtual Event"@en
AttendingVirtualMuseumTour	"Attending Virtual Museum Tour"@en
AttendingVirtualZooTour	"Attending Virtual Zoo Tour"@en
BPSDAssessmentTraining	"BPSD Assessment Training"@en
BalanceExercise	"Balance Exercise"@en
BalloonGame	"Balloon Game"@en
BarbecueActivity	"Barbecue Activity"@en
BehaviourTherapy	"Behaviour Therapy"@en
BibleReading	"Bible Reading"@en
BiographicallyOrientatedMobilisation	"Biographically Orientated Mobilisation"@en
BreathingExercise	"Breathing Exercise"@en
BrightWhiteLightTherapy	"Bright White Light Therapy"@en
BuddySystemIntervention	"Buddy System Intervention"@en
BusTrip	"Bus Trip"@en
CardGame	"Card Game"@en
CasinoActivity	"Casino Activity"@en
CelebrationActivity	"Celebration Activity"@en
ChangingContinenceAid	"Changing Continence Aid"@en

CheckingHearingAid	"Checking Hearing Aid"@en
CheckingPerson	"Checking Person"@en
CheckingVisionAid	"Checking Vision Aid"@en
ChurchServiceActivity	"Church Service Activity"@en
CleaningHearingAid	"Cleaning Hearing Aid"@en
CleaningVisionAid	"Cleaning Vision Aid"@en
CoffeeClubActivity	"Coffee Club Activity"@en
CognitiveBehaviouralTherapy	"Cognitive Behavioural Therapy"@en
CognitiveRehabilitation	"Cognitive Rehabilitation"@en
CognitiveStimulationTherapy	"Cognitive Stimulation Therapy"@en
CognitiveTherapy	"Cognitive Therapy"@en
CognitiveTraining	"Cognitive Training"@en
ColouringPicture	"Colouring Picture"@en
CommunicationSkillTraining	"Communication Skill Training"@en
ComputerisedCognitiveBehaviourTherapy	"Computerised Cognitive Behavioural Therapy"@en
ConsistencyOfStaff	"Consistency of Staff Providing Care"@en
Cooking	"Cooking"@en
Counselling	"Counselling"@en
CreativePursuitActivity	"Creative Pursuit Activity"@en
CrushingOralMedicationForPeopleWithSwallowingDifficulty	"Crushing Oral Medication for People with Swallowing Difficulty"@en
DanceTherapy	"Dance Therapy"@en
DeepBreathingExercise	"Deep Breathing Exercise"@en
Distraction	"Distraction"@en
DiversionalTherapy	"Diverstional Therapy"@en
DollTherapy	"Doll Therapy"@en
EmotionalSupport	"Emotional Support"@en
EmotionalAndMoodDisturbanceEducationToFamilyMember	"Emotional and Mood Disturbance Education to Family Member"@e
EncouragingCommunication	"Encouraging Communication"@en
EncouragingPhysicalActivity	"Encouraging Physical Activity"@en
EncouragingToDrinkWater	"Encouraging to Drink Water"@en
EncouragingToEatFood	"Encouraging to Eat Food"@en
EngagingPersonInActivity	"Engaging Person in Activity"@en
EnhancingVisualAccess	"Enhancing Visual Access"@en
EnvironmentalModification	"Environmental Modification"@en
Ergotherapy	"Ergotherapy"@en
ExplanationOfNursingCare	"Explanation of Nursing Care"@en
FamilyMemberFocusedIntervention	"Family Member Focused Intervention"@en
FamilyMemberVisit	"Family Member Visit"@en
FlexibilityExercise	"Flexibility Exercise"@en
FloorGame	"Floor Game"@en
FoldingClothes	"Folding Clothes"@en
FoldingTowel	"Folding Towel"@en
FootyTippingActivity	"Footy Tipping Activity"@en
FriendVisit	"Friend Visit"@en
Gardening	"Gardening"@en
GetIntoReading	"Get into Reading"@en
GivingEncouragement	"Giving Encouragement"@en
GolfPuttingGame	"Golf Putting Game"@en
HairAppointment	"Hair Appointment"@en
Handicraft	"Handicraft"@en
HappyHourActivity	"Happy Hour Activity"@en
HavingGarden	"Having Garden"@en
HavingGardenShed	"Having Garden Shed"@en
HavingKitchen	"Having Kitchen"@en
HavingPersonalFurnishing	"Having Personal Furnishing"@en
HavingPersonalMemoryBox	"Having Personal Memory Box"@en
HavingPersonalisedDoor	"Having Personalised Door"@en
HavingRegularRoutine	"Having Regular Routine"@en
HavingWashingLine	"Having Washing Line"@en
HealthCareProfessionalFocusedIntervention	"Health Care Professional Focused Intervention"@en
HealthCareProfessionalRecognitionProgram	"Health Care Professional Recognition Program"@en
Homeopathy	"Homeopathy"@en
HorticulturalTherapy	"Horticultural Therapy"@en

HumorTherapy	"Humor Therapy"@en
InformingLegalRepresentative	"Informing Legal Representative"@en
InformingMedicalPractitioner	"Informing Medical Practitioner"@en
InformingNurse	"Informing Nurse"@en
InformingRelative	"Informing Relative"@en
InteractionWithOtherCareRecipient	"Interaction with Other Care Recipient"@en
IntergenerationalCare	"Intergenerational Care"@en
InternetBasedIntervention	"Internet-Based Intervention"@en
InterpersonalPsychotherapy	"Interpersonal Psychotherapy"@en
JapaneseGardenIntervention	"Japanese Garden Intervention"@en
KnittingAcitivity	"Knitting Activity"@en
LegoActivity	"Lego Activity"@en
LiaisingWithLegalRepresentative	"Liaising with Legal Representative"@en
LiaisingWithMedicalPractitioner	"Liaising with Medical Practitioner"@en
LiaisingWithPharmacist	"Liaising with Pharmacist"@en
LiasingWithRelative	"Liaising with Relative"@en
LightTherapy	"Light Therapy"@en
ListeningToAudiobook	"Listening to Audiobook"@en
ListeningToClassicalMusic	"Listening to Classical Music"@en
ListeningToFamiliarMusic	"Listening to Familiar Music"@en
ListeningToPreferredMusic	"Listening to Preferred Music"@en
ListeningToRadio	"Listening to Radio"@en
ListeningToRelaxingMusic	"Listening to Relaxing Music"@en
LockingUpMedicationWhenNotInUse	"Locking Up Medication When not in Use"@en
MakingBed	"Making Bed"@en
MakingBuzzerEasyToReach	"Making Buzzer Easy to Reach"@en
MakingExitLessObvious	"Making Exit Less Obvious"@en
MassageTherapy	"Massage Therapy"@en
MatchingSocks	"Matching Socks"@en
MemoryTraining	"Memory Training"@en
MindBodyTherapy	"Mind-Body Therapy"@en
MiniMartActivity	"Mini Mart Activity"@en
Monitoring	"Monitoring"@en
MonitoringPersonTakingMedication	"Monitoring Person Taking Medication"@en
MontessoriIntervention	"Montessori Intervention"@en
MultisensoryStimulation	"Multisensory Stimulation"@en
MusicTherapeuticCaregiving	"Music Therapeutic Caregiving"@en
MusicTherapy	"Music Therapy"@en
NatureOfBPSDTraining	"Nature of BPSD Training"@en
NoiseControl	"Noise Control"@en
NonpharmacologicalIntervention	"Nonpharmacological Intervention"@en
NonpharmacologicalInterventionTraining	"Nonpharmacological Intervention Training"@en
NursingPracticeIntervention	"Nursing Practice Intervention"@en
Observation	"Observation"@en
OccupationalTherapy	"Occupational Therapy"@en
OfferingDrink	"Offering Drink"@en
OfferingFood	"Offering Food"@en
OneOnOnePersonalContact	"One on One Interaction"@en
PRIDEBathingApproach	"PRIDE Bathing Approach"@en
PaintingPicture	"Painting Picture"@en
PamperGroupActivity	"Pamper Group Activity"@en
ParticipatingActiveMusicTherapyByMovingBody	"Participating Active Music Therapy by Moving Body"@en
ParticipatingActiveMusicTherapyByPlayingMusicalInstrument	"Participating Active Music Therapy by Playing Musical Instrument"@
ParticipatingActiveMusicTherapyBySinging	"Participating Active Music Therapy by Singing"@en
ParticipatingActiveMusicTherapyByWritingSong	"Participating Active Music Therapy by Writing Song"@en
PassiveMusicTherapy	"Passive Music Therapy"@en
PastoralCare	"Pastoral Care"@en
PayingCloseAttentionToSharpObject	"Paying Close Attention to Sharp Object"@en
PersonCentredCareEudcationToHealthCareProfessional	"Person Centred Care Education to Health Care Professional"@en
PersonWithDementiaFocusedIntervention	"Person with Dementia Focused Intervention"@en
PetTherapy	"Pet Therapy"@en
PhoneCall	"Phone Call"@en
PhysicalEnvironmentalIntervention	"Physical Environmental Intervention"@en

PhysicalExerciseTherapy	"Physical Exercise Therapy"@en
PhysicalRestraint	"Physical Restraint"@en
Physiotherapy	"Physiotherapy"@en
PlayingBallGame	"Playing Ball Game"@en
PlayingBingoGame	"Playing Bingo Game"@en
PlayingBoardGame	"Playing Board Game"@en
PlayingBowling	"Playing Bowling"@en
PlayingCarpetBowels	"Playing Carpet Bowels"@en
PlayingChairVolleyball	"Playing Chair Volleyball"@en
PlayingVideoGame	"Playing Video Game"@en
PoetryTherapy	"Poetry Therapy"@en
PolishingFavouriteObject	"Polishing Favourite Object"@en
PoppingBubbleWrap	"Popping Bubble Wrap"@en
ProblemSolvingTherapy	"Problem Solving Therapy"@en
ProvidingActivityArea	"Providing Activity Area"@en
ProvidingAdequateRest	"Providing Adequate Rest"@en
ProvidingCaffeineFreeBeverage	"Providing Caffeine-Free Beverage"@en
ProvidingCalmEnvironment	"Providing Calm Environment"@en
ProvidingClearMarkedAccessToBathroom	"Providing Clear Marked Access to Bathroom"@en
ProvidingClearMarkedAccessToPersonalBelonging	"Providing Clear Marked Access to Personal Belonging"@en
ProvidingConcentratedOxygen	"Providing Concentrated Oxygen"@en
ProvidingContinenceAid	"Providing Continence Aid"@en
ProvidingCrashMatForBedsideSafety	"Providing Crash Mat for Bedside Safety"@en
ProvidingDryingRackInRoom	"Providing Drying Rack In Room"@en
ProvidingEasyAccessToOutsideSpace	"Providing Easy Access to Outside Space"@en
ProvidingFavouriteFood	"Providing Favourite Food"@en
ProvidingHoist	"Providing Hoist"@en
ProvidingHomeLikeEnvironment	"Providing Home Like Environment"@en
ProvidingPrivateBathroom	"Providing Private Bathroom"@en
ProvidingSafeOutsideSpace	"Providing Safe Outside Space"@en
ProvidingSensorMat	"Providing Sensor Mat"@en
ProvidingSingleBedroom	"Providing Single Bedroom"@en
ProvidingSnack	"Providing Snack"@en
ProvidingSufficientLighting	"Providing Sufficient Lighting"@en
ProvidingAVarietyOfSpaceInEnvironment	"Providing a Variety of Space in Environment"@en
Psychoeducation	"Psychoeducation"@en
Psychotherapy	"Psychotherapy"@en
ReadingActivity	"Reading Activity"@en
ReadingBook	"Reading Book"@en
ReadingMagazine	"Reading Magazine"@en
ReadingNewspaper	"Reading Newspaper"@en
RealityOrientationTherapy	"Reality Orientation Therapy"@en
Reassurance	"Reassurance"@en
RecreationalTherapy	"Recreational Therapy"@en
Redirection	"Redirection"@en
ReducingClutter	"Reducing Clutter"@en
ReducingLightGlare	"Reducing Light Glare"@en
ReducingNoiseOfNonemergencyOverheadPaging	"Reducing Noise of Non-emergency Overhead Paging"@en
ReductionOfDaytimeSleep	"Reduction of Daytime Sleep"@en
ReferralToPolice	"Referral to Police"@en
ReferralToPsychiatrist	"Referral to Psychiatrist"@en
ReferralToPsychogeriatration	"Referral to Psychogeriatration"@en
ReferralToPsychologist	"Referral to Psychologist"@en
ReferralToRequiredServiceProvider	"Referral to Required Service Provider"@en
ReferralToASpecialist	"Referral to a Specialist"@en
Reflexology	"Reflexology"@en
RegularToileting	"Regular Toileting"@en
RelaxationTherapy	"Relaxation Therapy"@en
RelocatingPersonToAppropriateArea	"Relocating Person to Appropirate Area"@en
Reminding	"Reminding"@en
ReminiscenceTherapy	"Reminiscence Therapy"@en
RemovingDangerousObject	"Removing Dangerous Object"@en
Reorientation	"Reorientation"@en

ReorientationToEvent	"Reorientation to Event"@en
ReorientationToPerson	"Reorientation to Person"@en
ReorientationToPlace	"Reorientation to Place"@en
ReorientationToTime	"Reorientation to Time"@en
Repositioning	"Repositioning"@en
RoboticAnimalAssistedTherapy	"Robotic Animal-Assisted Therapy"@en
ScheduledReassuranceTherapy	"Scheduled Reassurance Therapy"@en
SchedulingChallengingActivityWhenPersonIsAtHisBest	"Scheduling Challenging Activity When Person with Dementia is at H
SelfMaintenanceTherapy	"Self-Maintenance Therapy"@en
SensoryStimulationTherapy	"Sensory Stimulation Therapy"@en
SewingActivity	"Sewing Activity"@en
SimplifyingTask	"Simplifying Task"@en
SimulatedPresenceTherapy	"Simulated Presence Therapy"@en
SittingAndTalkingWithPerson	"Sitting and Talking with Person"@en
SmellingFlower	"Smelling Flower"@en
SnoezolenMultisensoryStimulationTherapy	"Snoezelen Multisensory Stimulation Therapy"@en
SocialEnvironmentalIntervention	"Social Environmental Intervention"@en
SocialInteractionIntervention	"Social Interaction Intervention"@en
SocialMediaVideoCall	"Social Media Video Call"@en
SocialOuting	"Social Outing"@en
SocialSupportGroupProgram	"Social Support Group Program"@en
SolvingWordSearchPuzzle	"Solving Word Search Puzzle"@en
SonasIntervention	"Sonas Intervention"@en
SortingButton	"Sorting Button"@en
SortingCoin	"Sorting Coin"@en
SpiritualSupport	"Spiritual Support"@en
SpiritualTherapy	"Spiritual Therapy"@en
StaffSupervision	"Staff Supervision"@en
Storytelling	"Storytelling"@en
StrengthExercise	"Strength Exercise"@en
StretchingExercise	"Stretching Exercise"@en
StructuredActivity	"Structured Activity"@en
SunlightTherapy	"Sunlight Therapy"@en
SupportToFamilyMember	"Support to Family Member"@en
SupportToHealthCareProfessional	"Support to Health Care Professional"@en
Swimming	"Swimming"@en
TaiChi	"Tai Chi"@en
TastingSweetFood	"Tasting Sweet Food"@en
TenderLovingCare	"Tender Loving Care"@en
FloorGame	"The game that can be played on floors, e.g., floor chess game."@e
TherapyForEmotionalAndMoodDisturbanceInDementia	"Therapy for Emotional and Mood Disturbance in Dementia"@en
TouchTherapy	"Touch Therapy"@en
ToyAnimalAssistedTherapy	"Toy Animal-Assisted Therapy"@en
TurningOffTelevision	"Turning off Television"@en
UsingCueCard	"Using Cue Card"@en
UsingSocialRobot	"Using Social Robot"@en
UsingUnobtrusiveSafetyDesignForPhysicalEnviornment	"Using Unobtrusive Safety Design for Physical Environment"@en
UsingVerbalReminder	"Using Verbal Reminder"@en
UsingWorryBeads	"Using Worry Beads"@en
UsingWrittenNoteAsReminder	"Using Written Note as Reminder"@en
UsingPortableBedsideCommode	"Using a Portable Bedside Commode"@en
ValidationTherapy	"Validation Therapy"@en
VirtualRealityTherapy	"Virtual Reality Therapy"@en
VolunteerProgram	"Volunteer Program"@en
Walking	"Walking"@en
WatchingMovie	"Watching Moive"@en
WatchingTelevision	"Watching Television"@en
WoodworkingActivity	"Woodworking Activity"@en
WorkforcePlanning	"Workforce Planning"@en
Yoga	"Yoga"@en

SPARQL query and query results for competency question No.8 “What background information of people with dementia is related to emotional and mood disturbance management in long-term care facilities?”

SPARQL query:

```
PREFIX rdf: <http://www.w3.org/1999/02/22-rdf-syntax-ns#>
PREFIX owl: <http://www.w3.org/2002/07/owl#>
PREFIX rdfs: <http://www.w3.org/2000/01/rdf-schema#>
PREFIX xsd: <http://www.w3.org/2001/XMLSchema#>
PREFIX :<http://www.semanticweb.org/zhenyuzhang/ontologies/2021/DREAMDNPTO#>
SELECT ?internationalised_resource_identifier_short_name_of_background_of_care_recipient ?label_of_background_of_care_recipient
            WHERE { ?internationalised_resource_identifier_short_name_of_background_of_care_recipient rdfs:subClassOf*
:BackgroundOfCareRecipient;
rdfs:label ?label_of_background_of_care_recipient. } ORDER BY ?label_of_background_of_care_recipient
```

internationalised_resource_identifier_short_name_of_background_of_care_recipient	label_of_background_of_care_recipient
AbilityToConcentrate	"Ability to Concentrate"@en
AbilityToIdentifyEvent	"Ability to Identify Event"@en
AbilityToIdentifyPerson	"Ability to Identify Person"@en
AbilityToIdentifyPlace	"Ability to Identify Place"@en
AbilityToIdentifyTime	"Ability to Identify Time"@en
AbilityToMakeDecision	"Ability to Make Decision"@en
AbilityToMobilise	"Ability to Mobilise"@en
AbilityToPerformActivityOfDailyLiving	"Ability to Perform Activity of Daily Living"@en
AbilityToReason	"Ability to Reason"@en
AbilityToTrustSomeone	"Ability to Trust Someone"@en
AboriginalPerson	"Aboriginal Person"@en
AcceptanceOfSupport	"Acceptance of Support"@en
Addiction	"Addiction"@en
Age	"Age"@en
AlcoholAddiction	"Alcohol Addiction"@en
Allergy	"Allergy"@en
AssociatedGroup	"Associated Group"@en
AssociatedProfessionalOrganization	"Associated Professional Organization"@en
AssociatedSocialClub	"Associated Social Club"@en
AtRiskState	"At Risk State"@en
AtRiskForFall	"At Risk for Fall"@en
AtRiskForPressureUlcer	"At Risk for Pressure Ulcer"@en
AtRiskForSuicide	"At Risk for Suicide"@en
AvoidantPersonalityTrait	"Avoidant Personality Trait"@en
BackgroundOfCareRecipient	"Background of Care Recipient"@en
BathPreference	"Bath Preference"@en
BeddingPreference	"Bedding Preference"@en
Belief	"Belief"@en
BiologicalGender	"Biological Gender"@en
BirdKeepingHobby	"Bird Keeping Hobby"@en
Bisexual	"Bisexual"@en
BodyHeight	"Body Height"@en
BodySize	"Body Size"@en
BodyWeight	"Body Weight"@en
BusinessImmigration	"Business Immigration"@en
CannabisAddiction	"Cannabis Addiction"@en
CatKeepingHobby	"Cat Keeping Hobby"@en
ChildhoodAbuse	"Childhood Abuse"@en
ClothingPreference	"Clothing Preference"@en
CognitiveAbility	"Cognitive Ability"@en
CognitiveStyle	"Cognitive Style"@en
ContactInformation	"Contact Information"@en
CookingHobby	"Cooking Hobby"@en
Coordination	"Coordination"@en
CountryOfBirth	"Country of Birth"@en
CraftHobby	"Craft Hobby"@en
CulturalBackground	"Culture Background"@en
DateOfBirth	"Date of Birth"@en

DeathOfCloseFriend	"Death of Close Friend"@en
DeathOfFamilyMember	"Death of Family Member"@en
DemographicInformation	"Demographic Information"@en
DogKeepingHobby	"Dog Keeping Hobby"@en
DomesticViolence	"Domestic Violence"@en
DrinkPreference	"Drink Preference"@en
DrugAllergy	"Drug Allergy"@en
EducationalStatus	"Educational Status"@en
EggAllergy	"Egg Allergy"@en
ElderAbuse	"Elder Abuse"@en
EmotionalDependency	"Emotional Dependency"@en
EmotionalReactivity	"Emotional Reactivity"@en
EthnicBackground	"Ethnic Background"@en
ExposureToCombat	"Exposure to Combat"@en
Extraversion	"Extraversion"@en
FamilyHistoryOfMentalDisorder	"Family History of Mental Disorder"@en
FamilyImmigration	"Family Immigration"@en
FamilyName	"Family Name"@en
FamilyRole	"Family Role"@en
Female	"Female"@en
FestivalPreference	"Festival Preference"@en
FirstGenerationImmigrant	"First Generation Immigrant"@en
FishAllergy	"Fish Allergy"@en
FishingHobby	"Fishing Hobby"@en
FishkeepingHobby	"Fishkeeping Hobby"@en
FoodAllergy	"Food Allergy"@en
FoodPreference	"Food Preference"@en
FootPreference	"Foot Preference"@en
GamblingAddiction	"Gambling Addiction"@en
GardeningHobby	"Gardening Hobby"@en
Gay	"Gay"@en
GenderIdentity	"Gender Identity"@en
GivenName	"Given Name"@en
HairCarePreference	"Hair Care Preference"@en
HallucinogenAddiction	"Hallucinogen Addiction"@en
HandPreference	"Hand Preference"@en
HappyLifeEvent	"Happy Life Event"@en
HearingAbility	"Hearing Ability"@en
Heterosexuality	"Heterosexuality"@en
HistoryOfArthritis	"History of Arthritis"@en
HistoryOfAsthma	"History of Asthma"@en
HistoryOfCancer	"History of Cancer"@en
HistoryOfChoking	"History of Choking"@en
HistoryOfChronicHealthCondition	"History of Chronic Health Condition"@en
HistoryOfChronicObstructivePulmonaryDisease	"History of Chronic Obstructive Pulmonary Disease"@en
HistoryOfConstipation	"History of Constipation"@en
HistoryOfDiabetes	"History of Diabetes"@en
HistoryOfFall	"History of Fall"@en
HistoryOfHeadInjury	"History of Head Injury"@en
HistoryOfHeartDisease	"History of Heart Disease"@en
HistoryOfHeartFailure	"History of Heart Failure"@en
HistoryOfHighCholesterol	"History of High Cholesterol"@en
HistoryOfHypertension	"History of Hypertension"@en
HistoryOfKidneyDisease	"History of Kidney Disease"@en
HistoryOfMedication	"History of Medication"@en
HistoryOfMentalHealth	"History of Mental Health"@en
HistoryOfObesity	"History of Obesity"@en
HistoryOfOralDisease	"History of Oral Disease"@en
HistoryOfOsteoporosis	"History of Osteoporosis"@en
HistoryOfPain	"History of Pain"@en
HistoryOfSleepDisturbance	"History of Sleep Disturbance"@en
HistoryOfTakingIllicitMedication	"History of Taking Illicit Medication"@en
HistoryOfTakingNonprescribedMedication	"History of Taking Non-prescribed Medication"@en

HistoryOfTakingPrescriptionMedication	"History of Taking Prescription Medication"@en
Hobby	"Hobby"@en
IllnessInFamilyMember	"Illness in Family Member"@en
ImmigrantGenerationStatus	"Immigrant Generation Status"@en
ImmigrationInformation	"Immigration Information"@en
ImmigrationType	"Immigration Type"@en
InformationAboutActivityOfDailyLiving	"Information about Activity of Daily Living"@en
InformationAboutContinenceCare	"Information about Continence Care"@en
InformationAboutEating	"Information about Eating"@en
InformationAboutMobility	"Information about Mobility"@en
InformationAboutPersonalGrooming	"Information about Personal Grooming"@en
InformationAboutPersonalHygiene	"Information about Personal Hygiene"@en
InformationAboutSleep	"Information about Sleep"@en
InsectAllergy	"Insect Allergy"@en
InternetGamingAddiction	"Internet Gaming Addiction"@en
Introversion	"Introversion"@en
KnittingHobby	"Knitting Hobby"@en
LGBTQ	"LGBTQ"@en
LanguageInformation	"Language Information"@en
LatexAllergy	"Latex Allergy"@en
Lesbian	"Lesbian"@en
LifeStyle	"Life Style"@en
ListeningToRadioHobby	"Listening to Radio Hobby"@en
Male	"Male"@en
Man	"Man"@en
MaritalStatus	"Marital Status"@en
MassagePreference	"Massage Preference"@en
MedicalHistory	"Medical History"@en
MedicareNumber	"Medicare Number"@en
MilkAllergy	"Milk Allergy"@en
MoldAllergy	"Mold Allergy"@en
MoralFramework	"Moral Framework"@en
MusicHobby	"Music Hobby"@en
NativeLanguage	"Native Language"@en
NeedForInterpreter	"Need for Interpreter"@en
NicotineAddiction	"Nicotine Addiction"@en
Nonbinary	"Non-Binary"@en
NumberOfChildren	"Number of Children"@en
OpioidAddiction	"Opioid Addiction"@en
PaintingHobby	"Painting Hobby"@en
PastOccupation	"Past Occupation"@en
PeanutAllergy	"Peanut Allergy"@en
PerceivedGender	"Perceived Gender"@en
PersonName	"Person Name"@en
PersonalHygienePreference	"Personal Hygiene Preference"@en
PersonalityTrait	"Personality Trait"@en
PetAllergy	"Pet Allergy"@en
PetKeepingHobby	"Pet Keeping Hobby"@en
PhysicalAbility	"Physical Ability"@en
PhysicalExerciseHobby	"Physical Exercise Hobby"@en
PlayingCardHobby	"Playing Card Hobby"@en
PoliticalRefugee	"Political Refugee"@en
PollenAllergy	"Pollen Allergy"@en
Preference	"Preference"@en
PreferredLanguage	"Preferred Language"@en
PreferredName	"Preferred Name"@en
PreviousLifeEvent	"Previous Life Event"@en
Queer	"Queer"@en
ReadingBookHobby	"Reading Book Hobby"@en
ReadingNewspaperHobby	"Reading Newspaper Hobby"@en
RefugeeImmigration	"Refugee Immigration"@en
RefugeeFromWarRelatedArea	"Refugee from War Related Area"@en
Relationship	"Relationship"@en

RelationshipWithFamilyMember	"Relationship with Family Member"@en
RelationshipWithFriend	"Relationship with Friend"@en
RelationshipWithPet	"Relationship with Pet"@en
Religion	"Religion"@en
ReligiousBelief	"Religious Belief"@en
ReligiousPracticeRoutine	"Religious Practice Routine"@en
RemainingAbility	"Remaining Ability"@en
RespectToOtherPerson	"Respect to Other Person"@en
RoleIdentity	"Role Identity"@en
RoutineOfAttendingReligiousService	"Routine of Attending Religious Service"@en
RoutineOfPraying	"Routine of Praying"@en
SecondGenerationImmigrant	"Second Generation Immigrant"@en
SensoryAbility	"Sensory Ability"@en
SeparationFromFamilyMember	"Separation from Family Member"@en
SexualAssault	"Sexual Assault"@en
SexualBehaviour	"Sexual Behaviour"@en
SexualOrientation	"Sexual Orientation"@en
Sexuality	"Sexuality"@en
ShavingPreference	"Shaving Preference"@en
ShellfishAllergy	"Shellfish Allergy"@en
SignLanguage	"Sign Language"@en

RelationshipWithFamilyMember	"Relationship with Family Member"@en
RelationshipWithFriend	"Relationship with Friend"@en
RelationshipWithPet	"Relationship with Pet"@en
Religion	"Religion"@en
ReligiousBelief	"Religious Belief"@en
ReligiousPracticeRoutine	"Religious Practice Routine"@en
RemainingAbility	"Remaining Ability"@en
RespectToOtherPerson	"Respect to Other Person"@en
RoleIdentity	"Role Identity"@en
RoutineOfAttendingReligiousService	"Routine of Attending Religious Service"@en
RoutineOfPraying	"Routine of Praying"@en
SecondGenerationImmigrant	"Second Generation Immigrant"@en
SensoryAbility	"Sensory Ability"@en
SeparationFromFamilyMember	"Separation from Family Member"@en
SexualAssault	"Sexual Assault"@en
SexualBehaviour	"Sexual Behaviour"@en
SexualOrientation	"Sexual Orientation"@en
Sexuality	"Sexuality"@en
ShavingPreference	"Shaving Preference"@en
ShellfishAllergy	"Shellfish Allergy"@en
SignLanguage	"Sign Language"@en

SkilledImmigration	"Skilled Immigration"@en
SmellAbility	"Smell Ability"@en
SocialHistory	"Social History"@en
SocialRole	"Social Role"@en
SocialisationHobby	"Socialisation Hobby"@en
SoybeanAllergy	"Soybean Allergy"@en
SpiritualBelief	"Spiritual Belief"@en
StimulantAddiction	"Stimulant Addiction"@en
SurvivingHolocaust	"Surviving Holocaust"@en
TasteAbility	"Taste Ability"@en
ToiletryPreference	"Toiletry Preference"@en
TorresStraitIslander	"Torres Strait Islander"@en
TouchAbility	"Touch Ability"@en
TowelPreference	"Towel Preference"@en
Transgender	"Transgender"@en
TraumaticLifeEvent	"Traumatic Life Event"@en
TreeNutAllergy	"Tree Nut Allergy"@en
VerbalCommunicationAbility	"Verbal Communication Ability"@en
VisionAbility	"Vision Ability"@en
VisitorPreference	"Visitor Preference"@en
WarExperience	"War Experience"@en
WatchingFootballGameHobby	"Watching Football Game Hobby"@en
WatchingSportShowHobby	"Watching Sport Show Hobby"@en
WatchingTennisGameHobby	"Watching Tennis Game Hobby"@en
WheatAllergy	"Wheat Allergy"@en
Woman	"Woman"@en

SPARQL query and query results for competency question No.9 “What factors affect the implementation of non-pharmacological interventions to emotional and mood disturbances of people with dementia living in long-term care facilities?”

SPARQL query:

```
PREFIX rdf: <http://www.w3.org/1999/02/22-rdf-syntax-ns#>
PREFIX owl: <http://www.w3.org/2002/07/owl#>
PREFIX rdfs: <http://www.w3.org/2000/01/rdf-schema#>
PREFIX xsd: <http://www.w3.org/2001/XMLSchema#>
PREFIX :<http://www.semanticweb.org/zhenyuzhang/ontologies/2021/DREAMDNPTO#>
SELECT ?internationalised_resource_identifier_short_name_of_factor ?label_of_factor
            WHERE { ?internationalised_resource_identifier_short_name_of_factor rdfs:subClassOf*
:FactorAffectingImplementationOfNonpharmacologicalIntervention;
rdfs:label ?label_of_factor. } ORDER BY ?label_of_factor
```

internationalised_resource_identifier_short_name_of_factor	label_of_factor
AvailabilityOfNonpharmacologicalInterventionImplementationResource	"Availability of Nonpharmacological Intervention Implementation Resource"@en
AvailabilityOfNonpharmacologicalInterventionTrainingResource	"Availability of Nonpharmacological Intervention Training Resource"@en
CareHoursPerCareRecipientDay	"Care Hours per Care Recipient Day"@en
FactorAffectingImplementationOfNonpharmacologicalIntervention	"Factor Affecting Implementation of Nonpharmacological Intervention"@en
NonpharmacologicalTreatmentKnowledgeOfHealthCareProfessional	"Nonpharmacological Treatment Knowledge of Health Care Professional"@en
OrganisationalCulture	"Organisational Culture"@en
PersonCentredOrganisationalCulture	"Person-Centred Organisational Culture"@en
PersonalityOfHealthCareProfessional	"Personality of Health Care Professional"@en
SkillsMix	"Skills Mix"@en
SocialSupport	"Social Support"@en
StaffCareRecipientRatio	"Staff Care Recipient Ratio"@en
StaffStability	"Staff Stability"@en
TaskFocusedOrganisationalCulture	"Task-Focused Organisational Culture"@en

SPARQL query and query results for competency question No.10 “Who is involved in emotional and mood disturbances management in dementia care in long-term care facilities?”

SPARQL query:

```
PREFIX rdf: <http://www.w3.org/1999/02/22-rdf-syntax-ns#>
PREFIX owl: <http://www.w3.org/2002/07/owl#>
PREFIX rdfs: <http://www.w3.org/2000/01/rdf-schema#>
PREFIX xsd: <http://www.w3.org/2001/XMLSchema#>
PREFIX :<http://www.semanticweb.org/zhenyuzhang/ontologies/2021/DREAMDNPTO#>
SELECT ?internationalised_resource_identifier_short_name_of_person ?label_of_person
            WHERE { ?internationalised_resource_identifier_short_name_of_person rdfs:subClassOf* :Person;
rdfs:label ?label_of_person. } ORDER BY ?label_of_person
```

internationalised_resource_identifier_short_name_of_person	label_of_person
Aromatherapist	"Aromatherapist"@en
ArtTherapist	"Art Therapist"@en
Audiologist	"Audiologist"@en
Aunt	"Aunt"@en
Brother	"Brother"@en
BrotherInLaw	"Brother-in-law"@en
Cardiologist	"Cardiologist"@en
CareCoordinator	"Care Coordinator"@en
CareRecipient	"Care Recipient"@en
CareStaff	"Care Staff"@en
Clinician	"Clinician"@en
Cousin	"Cousin"@en
CulturalSupportWorker	"Cultural Support Worker"@en
Daughter	"Daughter"@en
DaughterInLaw	"Daughter-in-law"@en
DeFactoPartner	"De Factor Partner"@en
Dentist	"Dentist"@en
Dietitian	"Dietitian"@en
EnrolledNurse	"Enrolled Nurse"@en
Exhusband	"Ex-husband"@en
Exwife	"Ex-wife"@en
ExercisePhysiologist	"Exercise Physiologist"@en
FamilyMember	"Family Member"@en
Father	"Father"@en
Friend	"Friend"@en
GeneralPractitioner	"General Practitioner"@en
Gerontologist	"Gerontologist"@en
Granddaughter	"Granddaughter"@en
Grandfather	"Grandfather"@en
Grandmother	"Grandmother"@en
Grandson	"Grandson"@en
GreatGranddaughter	"Great Granddaughter"@en
GreatGrandfather	"Great Grandfather"@en
GreatGrandmother	"Great Grandmother"@en
GreatGrandson	"Great Grandson"@en
HealthCareProfessional	"Health Care Professional"@en
Husband	"Husband"@en
Interpreter	"Interpreter"@en
LegalRepresentative	"Legal Representative"@en
MedicalPractitioner	"Medical Practitioner"@en
Mother	"Mother"@en
MusicTherapist	"Music Therapist"@en
Nephew	"Nephew"@en
Nephrologist	"Nephrologist"@en
Niece	"Niece"@en
Nurse	"Nurse"@en
NursePractitioner	"Nurse Practitioner"@en
NursingManager	"Nursing Manager"@en
NursingTeamLeader	"Nursing Team Leader"@en

Nutritionist	"Nutritionist"@en
OccupationalTherapist	"Occupational Therapist"@en
Optometrist	"Optometrist"@en
PastoralCounselor	"Pastoral Counselor"@en
Person	"Person"@en
PersonWithDementia	"Person with Dementia"@en
PersonWithoutDementia	"Person without Dementia"@en
Pharmacist	"Pharmacist"@en
PhysicalTherapist	"Physical Therapist"@en
Podiatrist	"Podiatrist"@en
Priest	"Priest"@en
Psychiatrist	"Psychiatrist"@en
Psychogeriatrician	"Psychogeriatrician"@en
Psychologist	"Psychologist"@en
RecreationalTherapist	"Recreational Therapist"@en
RegisteredNurse	"Registered Nurse"@en
Sister	"Sister"@en
SisterInLaw	"Sister-in-law"@en
SocialWorker	"Social Worker"@en
Son	"Son"@en
SonInLaw	"Son-in-law"@en
SonInLaw	"Son-in-law"@en
SpeechTherapist	"Speech Therapist"@en
Therapist	"Therapist"@en
Uncle	"Uncle"@en
Volunteer	"Volunteer"@en
Wife	"Wife"@en

SPARQL query and query results for competency question No.11 “What are the effects of emotional and mood disturbances in dementia in long-term care facilities?”

SPARQL query:

```
PREFIX rdf: <http://www.w3.org/1999/02/22-rdf-syntax-ns#>
PREFIX owl: <http://www.w3.org/2002/07/owl#>
PREFIX rdfs: <http://www.w3.org/2000/01/rdf-schema#>
PREFIX xsd: <http://www.w3.org/2001/XMLSchema#>
PREFIX :<http://www.semanticweb.org/zhenyuzhang/ontologies/2021/DREAMDNPTO#>
SELECT ?internationalised_resource_identifier_short_name_of_effect ?label_of_effect
        WHERE { ?internationalised_resource_identifier_short_name_of_effect rdfs:subClassOf*
:EffectOfEmotionalAndMoodDisturbanceInDementia;
rdfs:label ?label_of_effect. } ORDER BY ?label_of_effect
```

internationalised_resource_identifier_short_name_of_effect	label_of_effect
Absconding	"Absconding"@en
AffectingTimingOfCareDeliveryToOtherCareRecipient	"Affecting Timing of Care Delivery to Other Care Recipient"@en
Agitation	"Agitation"@en
Anger	"Anger"@en
AnxietyOfHealthCareProfessional	"Anxiety of Health Care Professional"@en
Arguing	"Arguing"@en
Biting	"Biting"@en
BodyWeightChange	"Body Weight Change"@en
BurnoutOfHealthCareProfessional	"Burnout of Health Care Professional"@en
CeaselessTalking	"Ceaseless Talking"@en
ChangeInAppetite	"Change in Appetite"@en
ChangeInSleepPattern	"Change in Sleep Pattern"@en
Complaining	"Complaining"@en
ConcernOfFamilyMember	"Concern of Family Member"@en
ConcernOfFriend	"Concern of Friend"@en
ConcernOfOtherCareRecipient	"Concern of Other Care Recipient"@en
ConstantManipulationOfObject	"Constant Manipulation of Object"@en
ConstantRepetitionOfWord	"Constant Repetition of Word"@en
ConstantUnwarrantedRequestsForAttention	"Constant Unwarranted Requests for Attention"@en
ConstantUnwarrantedRequestsForHelp	"Constant Unwarranted Requests for Help"@en
ConstantUnwarrantedRequestsForReassurance	"Constant Unwarranted Requests for Reassurance"@en
Cursing	"Cursing"@en
DecliningCare	"Declining Care"@en
DecliningToTakeMedication	"Declining to Take Medication"@en
DecreaseInAppetite	"Decrease in Appetite"@en
DecreasedActivityOfDailyLiving	"Decreased Activity of Daily Living"@en
DepressionOfHealthCareProfessional	"Depression of Health Care Professional"@en
DestroyingProperty	"Destroying Property"@en
DisorganizedSpeech	"Disorganized Speech"@en
DisruptingNormalRoutineOfHealthCareProfessional	"Disrupting Normal Routine of Health Care Professional"@en
DisruptiveVocalisation	"Disruptive Vocalisation"@en
DisturbingOtherCareRecipient	"Disturbing Other Care Recipient"@en
Echolalia	"Echolalia"@en
EffectOfEmotionalAndMoodDisturbanceInDementia	"Effect of Emotional and Mood Disturbance in Dementia"@en
EffectOfEmotionalAndMoodDisturbanceOnFamilyMember	"Effect of Emotional and Mood Disturbance on Family Member"@en
EffectOfEmotionalAndMoodDisturbanceOnFriend	"Effect of Emotional and Mood Disturbance on Friend"@en
EffectOfEmotionalAndMoodDisturbanceOnHealthCareProfessional	"Effect of Emotional and Mood Disturbance on Health Care Professio
EffectOfEmotionalAndMoodDisturbanceOnOtherCareRecipient	"Effect of Emotional and Mood Disturbance on Other Care Recipient"
EffectOfEmotionalAndMoodDisturbanceOnSelf	"Effect of Emotional and Mood Disturbance on Self"@en
EffectOfEmotionalAndMoodDisturbanceOnSociety	"Effect of Emotional and Mood Disturbance on Society"@en
EmotionalDistress	"Emotional Distress"@en
FailureOfRehabilitation	"Failure of Rehabilitation"@en
Fidgeting	"Fidgeting"@en
Fighting	"Fighting"@en
Frustration	"Frustration"@en
Grabbing	"Grabbing"@en
Groaning	"Groaning"@en
Grunting	"Grunting"@en
HandWringing	"Hand Wringing"@en

HarmToFamilyMember	"Harm to Family Member"@en
HarmToFriend	"Harm to Friend"@en
HarmToHealthCareProfessional	"Harm to Health Care Professional"@en
HarmToOtherCareRecipient	"Harm to Other Care Recipient"@en
HidingObject	"Hiding Object"@en
Hitting	"Hitting"@en
Hoarding	"Hoarding"@en
Howling	"Howling"@en
ImpairedSocialFunctioning	"Impaired Social Functioning"@en
InappropriateDressing	"Inappropriate Dressing"@en
InappropriateGesture	"Inappropriate Gesture"@en
InappropriateSexualBehaviour	"Inappropriate Sexual Behaviour"@en
InappropriateUndressing	"Inappropriate Undressing"@en
InappropriatelyHandlingObject	"Inappropriately Handling Object"@en
IncreaseInAppetite	"Increase in Appetite"@en
IncreasedDependenceOnOtherPeople	"Increased Dependence On Other People"@en
IncreasedEmergencyDepartmentAdmission	"Increased Emergency Department Admission"@en
IncreasedHealthCareCost	"Increased Health Care Cost"@en
IncreasedHealthCareUtilisation	"Increased Health Care Utilisation"@en
IncreasedHospitalAdmission	"Increased Hospital Admission"@en
IncreasedHospitalisation	"Increased Hospitalisation"@en
IncreasedRiskOfAccidentalInjury	"Increased Risk of Accidental Injury"@en
IncreasedRiskOfElderAbuse	"Increased Risk of Elder Abuse"@en
InterferingWithOtherPeopleBelonging	"Interfering with Other People Belonging"@en
Irritability	"Irritability"@en
Kicking	"Kicking"@en
MakingBizarreNoise	"Making Bizarre Noise"@en
MoodSwing	"Mood Swing"@en
Negativism	"Negativism"@en
Outburst	"Outburst"@en
Pacing	"Pacing"@en
PhysicallyAggressiveBehaviour	"Physically Aggressive Behaviour"@en
PhysicallyNonaggressiveBehaviour	"Physically Nonaggressive Behaviour"@en
PointingFinger	"Pointing Finger"@en
PoorHygiene	"Poor Hygiene"@en
Punching	"Punching"@en
Pushing	"Pushing"@en
RamblingSpeech	"Rambling Speech"@en
RapidCognitiveDecline	"Rapid Cognitive Decline"@en
RepetitivePhysicalMannerism	"Repetitive Physical Mannerism"@en
RepetitiveQuestioning	"Repetitive Questioning"@en
Restlessness	"Restlessness"@en
Roaring	"Roaring"@en
Rocking	"Rocking"@en
Rummaging	"Rummaging"@en
Scratching	"Scratching"@en
Screaming	"Screaming"@en
Searching	"Searching"@en
SelfHarmBehaviour	"Self Harm Behaviour"@en
SelfTalk	"Self Talk"@en
Shouting	"Shouting"@en
Shoving	"Shoving"@en
Slamming	"Slamming"@en
SpeakingInExcessivelyLoudVoice	"Speaking in Excessively Loud Voice"@en
Spitting	"Spitting"@en
Staring	"Staring"@en
StressOfHealthCareProfessional	"Stress of Health Care Professional"@en
TakingOtherPeopleBelonging	"Taking Other People's Belonging"@en
Tearing	"Tearing"@en
Threat	"Threat"@en
ThrowingObject	"Throwing Object"@en
UsingAbusiveLanguage	"Using Abusive Language"@en
UsingAccusatoryLanguage	"Using Accusatory Language"@en

UsingHostileLanguage	"Using Hostile Language"@en
UsingObsceneLanguage	"Using Obscene Language"@en
UsingProfaneLanguage	"Using Profane Language"@en
VerballyAggressiveBehaviour	"Verbally Aggressive Behaviour"@en
VerballyNonaggressiveBehaviour	"Verbally Nonaggressive Behaviour"@en
Wandering	"Wandering"@en
WeightGain	"Weight Gain"@en
WeightLoss	"Weight Loss"@en

SPARQL query and query results for competency question No.12 “What communication skills are used to communicate with people with dementia?”

SPARQL query:

```
PREFIX rdf: <http://www.w3.org/1999/02/22-rdf-syntax-ns#>
PREFIX owl: <http://www.w3.org/2002/07/owl#>
PREFIX rdfs: <http://www.w3.org/2000/01/rdf-schema#>
PREFIX xsd: <http://www.w3.org/2001/XMLSchema#>
PREFIX :<http://www.semanticweb.org/zhenyuzhang/ontologies/2021/DREAMDNPTO#>
SELECT ?internationalised_resource_identifier_short_name_of_skill ?label_of_skill
            WHERE { ?internationalised_resource_identifier_short_name_of_skill rdfs:subClassOf*
:SkillForCommunicatingWithPersonWithDementia;
rdfs:label ?label_of_skill. } ORDER BY ?label_of_skill
```

internationalised_resource_identifier_short_name_of_skill	label_of_skill
ActiveListening	"Active Listening"@en
AllowingEnoughTimeForResponding	"Allowing Enough Time for Responding"@en
AvoidArguingWithPersonWithDementia	"Avoid Arguing with Person with Dementia"@en
AvoidAskingComplexQuestion	"Avoid Asking Complex Question"@en
AvoidAskingPersonWithDementiaToTryHarder	"Avoid Asking Person with Dementia to Try Harder"@en
AvoidCorrectingPersonWithDementia	"Avoid Correcting Person with Dementia"@en
AvoidCriticisingPersonWithDementia	"Avoid Criticising Person with Dementia"@en
AvoidInterruptingWhenPersonWithDementiaIsTalking	"Avoid Interrupting When Person with Dementia is Talking"@en
AvoidMultipleStepRequest	"Avoid Multiple-Step Request"@en
AvoidNegativeWord	"Avoid Negative Word"@en
AvoidOfferingTooManyChoices	"Avoid Offering Too Many Choices"@en
AvoidOpenEndedQuestion	"Avoid Open-Ended Question"@en
AvoidOverexplaining	"Avoid Overexplaining"@en
AvoidScreaming	"Avoid Screaming"@en
AvoidTellingPersonWithDementiaThatHeIsWrong	"Avoid Telling Person with Dementia that He is Wrong"@en
AvoidUsingHarshTone	"Avoid Using Harsh Tone"@en
AvoidUsingSlang	"Avoid Using Slang"@en
BreakingDownLongSentenceIntoShortPhrase	"Breaking Down Long Sentence into Short Phrase"@en
CelebratingSmallSuccess	"Celebrating Small Success"@en
CommunicatingActionAvoided	"Communicating Action Avoided"@en
CommunicatingActionSuggested	"Communicating Action Suggested"@en
CommunicatingAtEyeLevel	"Communicating at Eye Level"@en
ExpressingGratitude	"Expressing Gratitude"@en
FindingAQuietAndComfortableSpotToCommunicateWithPersonWithDe	"Finding a Quiet and Comfortable Spot to Communicate with Person [illegible]
GivingSingleStepInstruction	"Giving Single Step Instruction"@en
GivingVisualCue	"Giving Visual Cue"@en
HavingPatience	"Having Patience"@en
HelpingPeopleFindWordsForSelfExpression	"Helping People with Dementia Find Words for Self Expression"@en
InformingPersonWithDementiaBeforeGoingIntoPersonalSpace	"Informing Person with Dementia Before Going into Personal Space"@
IntroducingSelfForPeopleWhoAreUnableToRememberName	"Introducing Self for People Who are Unable to Remember Name"@e[illegible]
OfferingTwoChoices	"Offering Two Choices"@en
RephrashingUnclearVerbalCommunication	"Rephrashing Unclear Verbal Communication"@en
SkillForCommunicatingWithPersonWithDementia	"Skill for Communicating with Person with Dementia"@en
SpeakingClearly	"Speaking Clearly"@en
SpeakingDirectlyToPersonWithDementia	"Speaking Directly to Person with Dementia"@en
SpeakingSlowly	"Speaking Slowly"@en
TouchingGently	"Touching Gently"@en
UsingCalmTone	"Using Calm Tone"@en
UsingMinimalEncourager	"Using Minimal Encourager"@en
UsingNonverbalCommunication	"Using Nonverbal Communication"@en
UsingPleasantTone	"Using Pleasant Tone"@en
UsingPositiveWord	"Using Positive Word"@en
UsingSimpleLanguage	"Using Simple Language"@en
